# The survival impact of adjuvant radiotherapy and chemotherapy in patients with non-endometrioid endometrial carcinomas: a PSM-IPTW analysis based on SEER database

**DOI:** 10.1186/s12905-023-02429-6

**Published:** 2023-05-20

**Authors:** Zhimin Hao, Yangli Yu

**Affiliations:** The First Affiliated Hospital of Ningbo University, Ningbo, 315020 China

**Keywords:** Serous carcinoma, Carcinosarcoma, Clear cell carcinoma, Radiotherapy, Chemotherapy, IPTW

## Abstract

**Purpose:**

To investigate outcomes of adjuvant treatments for non-endometrioid endometrial carcinomas (NEEC), as previous studies are limited by its rarity and heterogeneity.

**Patients and methods:**

Patients with endometrial serous carcinoma (SC), clear cell carcinoma (CCC) and carcinosarcoma were identified between 2004 and 2018 from SEER database. Propensity score matching (PSM) along with inverse probability treatment weighting (IPTW) technique were employed to balance confounding factors. Multivariate, exploratory subgroup and sensitivity analyses were conducted to evaluate the impact of adjuvant treatment on overall survival (OS) and cause-specific survival (CSS).

**Results:**

The cohort comprised 5577 serous, 977 clear cell, and 959 carcinosarcomas. Combined chemotherapy and radiotherapy (CRT), chemotherapy alone, and radiotherapy alone were respectively administered in 42.21%, 47.27% and 10.58% of the whole cohort. Prior to adjusting, chemotherapy plus brachytherapy yielded the most beneficial effect among various strategies. After PSM-IPTW adjustment, CRT still demonstrated beneficial effect on OS and CSS. Subgroup analysis indicated CRT improved survival among various TNM stages, particularly with uterine carcinosarcoma. In the sensitivity analyses for serous histology, brachytherapy with or without chemotherapy appeared to benefit stage I-II patients. In stage III-IV SC patients, chemotherapy plus brachytherapy was still associated with improved survival outcomes. When nodal metastases were identified, additional external beam radiotherapy (EBRT) to CT was more utilized with survival improvement.

**Conclusion:**

In NEEC patients, combined CRT yielded beneficial effects than any single mode. Both chemotherapy and brachytherapy promoted survival in early stage SC patients. Late stage SC patients may benefit from chemotherapy plus either EBRT or brachytherapy.

**Supplementary Information:**

The online version contains supplementary material available at 10.1186/s12905-023-02429-6.

## Introduction

Non-endometrioid endometrial carcinomas (NEEC), which comprises approximately 20% of endometrial cancer, is classified to different subtypes with endometrial serous carcinoma (SC) being the most common, followed by clear-cell carcinoma (CCC) and uterine carcinosarcoma (UCS) [1]. SC and CCC subtypes constitute 40% and 8% of EC-related death, respectively [2]. UCS, due to its aggressive behavior, is recently lumped together with SC and CCC [3]. Currently, the gold standard of therapy comprises extrafascial hysterectomy with bilateral salpingo-oophorectomy (BSO) as well as appropriate adjuvant therapy. Given the high recurrence rate and poor prognosis, there is an increasing unmet need to identify the most appropriate adjuvant therapy for NEEC patients. However, clinical trials with regard to comparison between adjuvant chemotherapy (CT) and radiotherapy (RT) have not demonstrated significant difference in survival outcomes [4].

Periodically, pelvic or abdominal external beam radiotherapy (EBRT) has been the routine adjuvant treatment for women with high-risk endometrial cancer, although limited evidence on improving survival [5]. Compared to EBRT, vaginal brachytherapy is associated with better prognosis given its superiority of minimal side effects [6]. In contrast, with the purpose of reducing the incidence of distant metastases, CT was generally administered for EC patients with high risk factors; conversely; the increased locoregional recurrence rate after adjuvant chemotherapy alone preceded subsequent distant metastases and final death [7]. Thus, these differing patterns of treatment failure and side effects prompted more attempts to reduce local–regional and distant recurrences. Nevertheless, due to the rarity and heterogeneity of NEEC, current recommendations are still derived from existing experience of endometrioid endometrial carcinoma. More recently, results of three large randomized trials (GOG-249, GOG-258, and PORTEC-3) have been published. Among three above reports, endometrial serous and clear cell cancers in combination comprised only 29%, 19.3%, 20.8% of the whole patients enrolled, respectively [8–10]. Their relatively small percentage limited the possibility to draw robust conclusions via subset analysis. Also, UCS was not enrolled in the abovementioned randomized prospective trials. The optimal adjuvant treatment of NEEC has not been finally confirmed, and thus national guidelines such as the National Comprehensive Cancer Network (NCCN) allow variability in treatment. Of note, the potential survival benefit with combined modality treatment should be weighed against the cost of longer treatment duration, therapy-related severe adverse effects and impact on health-related quality of life.

Given these uncertainties, we analyzed nationwide patterns of survival outcomes of adjuvant CT and/or RT in women who underwent hysterectomy-based surgery for endometrial serous carcinoma, clear cell carcinoma and carcinosarcoma. The Surveillance, Epidemiology and End Results (SEER) database was selected given its large sample size as well as availability of adjuvant therapy and survival information.

## Materials and methods

### Study population

We conducted a retrospective analysis for patients with endometrial cancer of predominantly or purely serous, clear cell or carcinosarcoma histology. SEER database (SEER*Stat 8.3.9.2), which contains data of cancer patients from 18 regional registries (https://seer.cancer.gov/seerstat/), was employed for the analysis. We queried the 2020 release of SEER database from 2004, when modern staging information became available in SEER. Endometrial cancer was confirmed by histology of hysterectomy specimen and based on the WHO International Classification of Diseases for Oncology, third edition (ICD-O-3) morphology codes as follows: 8441-serous cystadenocarcinoma, NOS, 8460-papillary serous cystadenocarcinoma, 8461-serous surface papillary carcinoma; 8005-malignant tumor, clear cell type, 8310-clear cell adenocarcinoma, NOS; 8980–3-carcinosarcoma, NOS, 8981-carcinosarcoma, embryonal. Based on site-specific surgery codes, women who underwent at least total hysterectomy with or without bilateral salpingo-oophorectomy (site-specific surgery codes 40–77) were selected, including those with modified or radical hysterectomy. Receipt of adjuvant chemotherapy and/or radiotherapy after surgery was the focus of our study. Since all data included in the SEER database is publicly available online, this study does not require Institutional Review Board approval, or informed consent by the study subjects. While, we obtained permission to access the SEER program data from the US National Cancer Institute (reference number: 22756-Nov2020).

The exclusion criteria were listed as follows: (i) those cases with more than one malignancy or secondary tumor; (ii) missing information on patients’ age, cancer stage or survival period; (iii) those cases with the surgery code “local tumor excision or destruction; subtotal hysterectomy; surgery NOS’’ were excluded, given the fact that we could not identify the scope of the surgical procedure performed. (iv) cases without adjuvant therapy before or after hysterectomy were excluded. A landmark survival time of 3 months was applied in order to account for immortal time bias. These procedures were demonstrated as detailed in the supplementary Figure 1.

### Variable record and cohort definition

Demographic information of the patients encompassed age (< 50, 50–60, > 60), year of diagnosis (2004–2008, 2009–2013, 2014–2018), marital status (married, single, divorced/separated, widowed), race (white, black, others), and median household income. Tumor characteristics included histology subtypes (serous, clear cell and carcinosarcoma), TNM stage (T1N0M0, T2N0M0, T3-4aN0M0, TanyN1M0, TanyN2M0,TanyNanyM1), grade (grade I, well differentiated; grade II, moderately differentiated; grade III, poorly differentiated; grade IV, undifferentiated; unknown grade), tumor size (< 20, 20–39, 40–59, 60–79, > 80 mm, unknown). The tumor–node–metastasis (TNM) system of the American Joint Committee on Cancer was used in conjunction with FIGO staging [1]. Treatment data involved surgery mode (hysterectomy, extended hysterectomy), lymphadenectomy (yes, no or sentinel lymph node biopsy/removed), adjuvant therapy (RT alone, CT alone, CRT). Radiotherapy was subsequently divided to EBRT, VBT or combined of both.

### Outcome measures

Cause-specific survival (CSS) and overall survival (OS) were evaluated for outcome analysis. CSS was defined as the interval from final diagnosis to death due to endometrial cancer. The definition of OS was the time from confirmed diagnosis to death for any cause or to date of last follow-up. Patients who were alive at the last follow-up were censored.

### Statistical analysis

Categorical variables are illustrated as frequency and continuous variables are described as median (interquartile range [IQR]). Baseline patient characteristics were compared both pre- and post-matching with Chi-square test analysis, when the statistical significance in proportions’ differences with *p* value < 0.05 was considered unbalanced. To explore the effect of adjuvant therapy on survival in NEEC patients, multiple imputations by chained equations were performed to decrease potential bias due to missing data. First, we used a propensity score adjustment by inverse probability of treatment-weighting (IPTW) to maximally reduce the differences between radiotherapy and no radiotherapy administration, as previously described [11]. Specifically, the propensity score was calculated using a logistic regression model based on the abovementioned characteristics. Stratified by radiotherapy administrated or not, propensity score matching (PSM) method [12] was employed through the nearest neighbor-matching with caliper value 0.4 for 1:4 matching. Afterwards, IPTW was calculated as 1/PS in the group of radiotherapy given, whereas 1/ (1-PS) in the cohort without radiotherapy administered [13]. Stabilization of the IPTW was performed by multiplying the standard IPTW by the probability of undergoing treatment that each patient received [14]. Prior to and after IPTW-adjustment, univariate analysis (UVA) of patient characteristics effect on CSS and OS was conducted using the Kaplan–Meier (KM) method, with the log-rank method for evaluation for significance. Multivariable analysis (MVA) was performed through Cox proportional hazards regression model. Covariates enrolled in the MVA model were selected if they were significant in the UVA model. Next, we conducted exploratory subgroup analyses and evaluated heterogeneity as the subgroups are presumed to have been subjected to similar conditions. Quantification of heterogeneity was evaluated with the I2 statistic and the Cochran Q test. Random-effects models were used when study heterogeneity was high (I2 > 50%) and fixed-effects models were employed whereas heterogeneity was low (I2 ≤ 50%) [15]. In addition, we conducted the sensitivity analysis by comparing the CSS and OS for patients in different subgroup population and subgroup analysis. Finally, Kaplan–Meier plots illustrated CSS and OS rates based on adjuvant treatment administration in selected subgroups. Statistical analyses were executed with SPSS (version 22.0, SPSS, Chicago, IL, USA), R software (version 3.6.3; http://www.r-project.org/) and STATA-MP (version 17.0, College Station, TX, USA), with two-sided *P* < 0.05 considered statistically significant.

## Results

### Descriptive characteristics of the study population and survival outcome among all subgroups

According to the set criteria, a total of 7513 patients, who were diagnosed as NEEC as the primary malignancy and underwent at least total hysterectomy with adjuvant therapy administration, were extracted during 2010 and 2018 period. The median age at initial diagnosis was 66 years old [interquartile range (IQR): 61‐72 years old]. The median follow‐up period was 31 months [interquartile range (IQR): 18‐57 months]. The cohort comprised 5577 serous, 977 clear cell, 959 carcinosarcomas. Total hysterectomy with or without bilateral salping-oopharectomy was the main option for 90% (6764/7513) of cases, the remaining was concluded as extensive surgeries, including radical hysterectomy, pelvic exenteration or modified radical hysterectomy; meanwhile, lymphadenectomy was performed in 72.53% of patients. CRT was administered in 42.21% (3171/7513) of patients, similar to CT alone (47.27%), yet significantly higher than RT alone group (10.58%). The demographic and clinical characteristics of these NEEC patients and survival outcomes in those subgroups were summarized in Table 1.

**Table 1:** Univariate and multivariate analysis of predicting CSS and OS before IPTW-adjustment in NEEC patients

		**Cause-specific survival**				**Overall survival**			
		Univariate analysis		Multivariate analysis		Univariate analysis		Multivariate analysis	
Characteristics	Number	HR (95% CI)	*P*	HR (95% CI)	*P*	HR (95% CI)	*P*	HR (95% CI)	*P*
Age group (years)									
< 50	207	Reference				Reference			
50–65	3227	1.05 (0.83–1.32)	0.708	1.27 (1.0–1.61)	0.049	1.08 (0.86–1.35)	0.522	1.30 (1.04–1.63)	**0.022**
> 65	4079	1.24 (0.99–1.57)	0.067	**1.55 (1.22–1.96)**	** < 0.001**	1.40 (1.13–1.75)	**0.003**	1.71 (1.37–2.15)	** < 0.001**
Year of diagnosis									
2004–2008	1151	Reference				Reference			
2009–2013	2039	0.86 (0.78–0.95)	**0.003**	0.882(0.80–0.98)	**0.018**	0.86 (0.79–0.95)	**0.002**	0.90 (0.82–0.98)	0.029
2014–2018	4323	0.77 (0.70–0.85)	** < 0.001**	0.76(0.68–0.85)	** < 0.001**	0.77 (0.69–0.84)	** < 0.001**	0.78 (0.70–0.86)	** < 0.001**
Race recode									
Black	1616	Reference				Reference			
Others^a^	725	0.71 (0.61–0.83)	** < 0.001**	0.66 (0.56–0.77)	** < 0.001**	0.74 (0.64–0.85)	** < 0.001**	0.71 (0.61–0.81)	** < 0.001**
White	5172	0.84 (0.77–0.92)	** < 0.001**	0.82 (0.75–0.90)	** < 0.001**	0.85 (0.78–0.92)	** < 0.001**	0.84 (0.77–0.91)	** < 0.001**
Marital status									
Divorced/separated	956	Reference				Reference			
Married	3769	0.88 (0.79–0.99)	**0.032**	0.98 (0.87–1.09)	0.670	0.87 (0.78–0.96)	**0.008**	0.96 (0.86–1.07)	0.421
Single/unmarried	1209	1.02 (0.89–1.17)	0.782	0.97 (0.85–1.11)	0.632	1.00 (0.88–1.14)	0.999	0.96 (0.85–1.09)	0.560
Unknown	342	0.77 (0.62–0.95)	**0.014**	0.87 (0.69–1.08)	0.196	0.82 (0.67–0.99)	**0.044**	0.91 (0.75–1.11)	0.363
Widowed	1237	1.13 (0.99–1.29)	0.076	1.05 (0.92–1.19)	0.507	1.22 (1.08–1.37)	**0.002**	1.10 (0.97–1.25)	0.123
Median household income									
< $50,000	894	Reference				Reference			
$50,000–65,000	2135	0.99 (0.88–1.13)	0.955	1.10 (0.97–1.24)	0.136	0.96 (0.86–1.08)	0.483	1.06 (0.94–1.18)	0.347
> $65,000	4484	0.88 (0.78–0.98)	**0.025**	0.98 (0.87–1.10)	0.750	0.83 (0.75–0.92)	** < 0.001**	0.93 (0.83–1.03)	0.158
Grade									
I	103	Reference				Reference			
II	278	1.15 (0.73–1.81)	0.545	1.12 (0.71–1.76)	0.634	1.06 (0.72–1.56)	0.766	1.05 (0.71–1.54)	0.814
III	3523	1.65 (1.10–2.47)	**0.015**	1.42 (0.95–2.13)	0.090	1.33 (0.95–1.87)	0.101	1.18 (0.84–1.66)	0.340
IV	1846	1.63 (1.08–2.44)	**0.019**	1.37(0.91–2.07)	0.128	1.33 (0.95–1.88)	0.099	1.17 (0.83–1.65)	0.386
Unknown	1763	1.46 (0.97–2.20)	0.068	1.21 (0.81–1.83)	0.357	1.22 (0.86–1.72)	0.263	1.05 (0.74–1.48)	0.802
Histology									
Clear cell	977	Reference				Reference			
Serous	5577	1.23 (1.09–1.37)	** < 0.001**	1.11 (0.98–1.25)	0.093	1.17 (1.06–1.30)	**0.003**	1.10 (0.99–1.22)	0.087
Carcinosarcoma	959	1.99 (1.72–2.32)	** < 0.001**	2.02 (1.72–2.38)	** < 0.001**	1.90 (1.65–2.19)	** < 0.001**	1.98 (1.70–2.30)	** < 0.001**
TNM stage									
T1N0M0	2692	Reference				Reference			
T2N0M0	595	2.18 (1.82–2.61)	** < 0.001**	2.06 (1.72–2.47)	** < 0.001**	2.02 (1.72–2.36)	** < 0.001**	1.92 (1.63–2.25)	** < 0.001**
T3-4aN0MO	919	3.58 (3.11–4.12)	** < 0.001**	3.28 (2.84–3.80)	** < 0.001**	3.02 (2.67–3.43)	** < 0.001**	2.83 (2.49–3.23)	** < 0.001**
TanyN1M0	1372	4.04 (3.55–4.59)	** < 0.001**	3.97 (3.46–4.56)	** < 0.001**	3.48 (3.11–3.90)	** < 0.001**	3.56 (3.15–4.03)	** < 0.001**
TanyN2M0	258	5.18 (4.12–6.51)	** < 0.001**	4.84 (3.81–6.16)	** < 0.001**	4.26 (3.40–5.26)	** < 0.001**	4.08 (3.24–5.13)	** < 0.001**
TanyNanyM1	1677	9.95 (8.85–11.19)	** < 0.001**	7.81 (6.83–8.92)	** < 0.001**	8.07 (7.27–8.95)	** < 0.001**	6.52(5.78–7.35)	** < 0.001**
Surgery mode									
Total hysterectomy	6764	Reference				Reference			
Extendedl hysterectomy	749	1.50 (1.35–1.68)	** < 0.001**	1.12 (1.00–1.25)	0.046	1.45 (1.31–1.61)	** < 0.001**	1.10 (0.99–1.21)	0.086
Lymphadenectomy									
Yes	5449	Reference				Reference			
No	1658	2.30 (2.13–2.49)	** < 0.001**	1.46 (1.33–1.59)	< 0.001	2.23 (2.07–2.41)	** < 0.001**	1.47 (1.35–1.60)	** < 0.001**
SLN biopsy/removed	406	0.89 (0.72–1.10)	0.285	1.03 (0.83–1.29)	0.762	0.88 (0.71–1.08)	0.205	1.01 (0.82–1.24)	0.931
Tumor Size (mm)									
< 20	905	Reference				Reference			
20–39	1639	1.38 (1.18–1.61)	** < 0.001**	1.13 (0.97–1.32)	0.111	1.37 (1.19–1.58)	** < 0.001**	1.15 (0.99–1.33)	0.057
40–59	1494	1.82 (1.56–2.12)	** < 0.001**	1.31 (1.12–1.53)	** < 0.001**	1.76 (1.52–2.02)	** < 0.001**	1.29 (1.11–1.48)	** < 0.001**
60–79	808	2.36(2.00–2.78)	** < 0.001**	1.33 (1.12–1.57)	**0.001**	2.33 (1.99–2.71)	** < 0.001**	1.35 (1.15–1.58)	** < 0.001**
≥ 80	778	3.05 (2.59–3.59)	** < 0.001**	1.46 (1.23–1.73)	** < 0.001**	2.97 (2.55–3.46)	** < 0.001**	1.49 (1.28–1.75)	** < 0.001**
Unknown	1889	1.88 (1.62–2.17)	** < 0.001**	1.24(1.07–1.43)	**0.005**	1.85 (1.61–2.11)	** < 0.001**	1.26 (1.10–1.45)	** < 0.001**
Adjuvant therapy									
CRT	3171	Reference				Reference			
RT alone	795	0.94 (0.81–1.08)	0.381			1.12 (0.99–1.27)	0.066	1.61 (1.41–1.84)	** < 0.001**
CT alone	3547	1.93 (1.78–2.09)	** < 0.001**			1.88 (1.74–2.02)	** < 0.001**	1.26 (1.16–1.37)	** < 0.001**
Subclassification of AT									
CT + EBRT	1236	Reference				Reference			
EBRT + VBT	153	0.64 (0.47–0.86)	0.004	0.99 (0.72–1.35)	0.947	0.74 (0.57–0.97)	0.027	1.08 (0.83–1.42)	0.564
CT alone	3547	1.39 (1.26–1.54)	< 0.001	1.15 (1.03–1.29)	**0.010**	1.38 (1.25–1.52)	** < 0.001**	1.17 (1.06–1.30)	**0.003**
EBRT alone	336	0.96 (0.79–1.16)	0.661	1.60 (1.31–1.96)	** < 0.001**	1.13 (0.95–1.33)	0.168	1.77 (1.48–2.12)	** < 0.001**
VBT alone	306	0.43 (0.33–0.56)	< 0.001	1.15 (0.87–1.52)	0.322	0.57(0.455–0.709)	** < 0.001**	1.36 (1.08–1.72)	**0.009**
CT + VBT	1403	0.46 (0.39–0.53)	< 0.001	0.84 (0.72–0.98)	**0.024**	0.48 (0.42–0.55)	** < 0.001**	0.84 (0.73–0.97)	**0.018**
CT + EBRT + VBT	532	0.88 (0.74–1.05)	0.148	0.89(0.75–1.06)	0.184	0.89 (0.76–1.04)	0.152	0.90 (0.77–1.06)	0.202

*CT* chemotherapy, *RT* radiotherapy, *CRT* chemoradiotherapy, *AT* adjuvant therapy, *SLN* sentinel lymph node. Race Others^a^: American Indian, Asian/Pacific Islander

The effect of various characteristics on CSS and OS were evaluated using the KM method. In the univariable survival analysis (Table 1), significantly poorer CSS and OS were observed with increasing cancer stage and tumor size (*p* < 0.001). Other factors associated with worse CSS and OS included histology of carcinosarcoma and serous type, extensive surgeries, no performance of lymphadenectomy, low household income and year of diagnosis between 2004 and 2009. Patients with white race composed of the large proportion in the whole cohort and posed better CSS and OS outcomes than those of black race. Patients older than 65 years demonstrated poor OS outcome than those younger, although no CSS difference in the age group. Regarding adjuvant treatment, CT alone provided poorer survival impact compared to those CRT cases (HR = 1.929, *P* < 0.001), however, similar survival outcome was observed between RT alone and CRT group (HR = 0.938, *P* > 0.05). More importantly, CT plus VBT deserved the most beneficial effect on CSS and OS. Besides, tumor grade was not evidently associated with prognosis. In multivariable analysis with correction for other covariates (Table 1), increasing tumor size and patients’ age, progression of disease stage, no procedure of lymphadenectomy, and histology of carcinosarcoma were still related to poor survival. However, there was no statistical difference in serous and clear cell type for both CSS and OS. In comparison to CT or RT alone, receipt of CT plus VBT was associated with CSS and OS benefit (all *P* < 0.001, HR > 1). Other covariates, such as tumor grade, marital status, surgery mode and household income were not statistically associated with survival outcome.

### Exploration of adjuvant CRT utilization and RT/CT alone among subgroups

To further explore the association of adjuvant therapy among various clinicopathologic parameters, we stratified the cohort by receipt of adjuvant CRT, RT or CT alone. Before PSM and IPTW-adjustment by adjuvant therapy, most baseline characteristics were significantly unbalanced. Patients who received CRT tended to be aged between 50 and 65, diagnosed between 2014 and 2018, with histology of carcinosarcoma and serous type, in groups of advanced stage and tumor size bigger than 40 mm. Compared to CT alone, CRT administration was more common in patients who were diagnosed in recent period and as carcinosarcoma, in various cancer stage except distant metastasis (TanyNanyM1). After PSM and IPTW-adjustment by CRT *vs.* RT alone and CRT *vs.* CT alone respectively, all baseline characteristics were well balanced with *P* > 0.05. The results were demonstrated in supplementary Table 1. Thus both cohorts were explored for further analysis.

### Univariate and multivariate analysis for cause-specific survival and overall survival

After PSM and IPTW-adjustment, receipt of RT alone showed similar CSS and OS outcome compared to CRT based on univariate analysis (UVA), whereas, detrimental CSS (HR 1.475, 95% CI 1.252–1.736) and OS (HR 1.637, 95% CI 1.421–1.886) outcome on multivariate analysis (MVA), both with statistical significance as listed in Table 2. As described above, CT alone showed detrimental effect compared to CRT on CSS and OS (HR > 1, *P* < 0.001) both on UVA and MVA. Similar results were obtained following PSM and IPTW-adjustment, as shown in Table 3. CSS and OS improvements in patients who underwent RT persisted, as did the CSS and OS detriments associated with all other significant factors pre-adjustment.

**Table 2:** Survival analysis of predicting CSS and OS after IPTW-adjusted by CRT or RT in NEEC patients

	**Cause-specific survival**				**Overall survival**			
	Univariate analysis		Multivariate analysis		Univariate analysis		Multivariate analysis	
Characteristics	HR (95% CI)	*P*	HR (95% CI)	*P*	HR (95% CI)	*P*	HR (95% CI)	*P*
Adjuvant treatment								
CRT	Reference				Reference			
RT alone	0.94 (0.82–1.81)	0.384	1.48 (1.25–1.74)	< 0.001	1.11 (0.98–1.26)	0.104	1.64 (1.42–1.89)	< 0.001
Age group (years)								
< 50	Reference				Reference			
50–65	1.03 (0.70–1.49)	0.896	0.99 (0.68–1.46)	0.981	0.93 (0.65–1.34)	0.707	1.15 (0.80–1.65)	0.459
> 65	1.29 (1.15–1.46)	< 0.001	1.32 (0.90–1.94)	0.153	1.40 (1.26–1.56)	0.024	1.60 (1.11–2.30)	0.011
Year of diagnosis								
2004–2008	Reference				Reference			
2009–2013	0.90 (0.77–1.05)	0.164	0.91 (0.78–1.08)	0275	0.88 (0.76–1.01)	0.061	0.92 (0.80–1.07)	0.271
2014–2018	0.85 (0.73–0.99)	0.036	0.87 (0.73–1.03)	0.114	0.82 (0.71–0.94)	0.005	0.87 (0.74–1.02)	0.093
Race redode								
Black	Reference				Reference			
Others^a^	0.64 (0.49–0.83)	0.001	0.58 (0.45–0.76)	< 0.001	0.71 (0.56–0.89)	0.004	0.69 (0.54–0.88)	0.002
White	0.84 (0.73–0.97)	0.015	0.83 (0.71–0.96)	0.010	0.86 (0.75–0.97)	0.017	0.85 (0.75–0.98)	0.023
Marital status								
Divorced/separated	Reference				Reference			
Married	0.92 (0.77–1.10)	0.347	1.03 (0.85–1.23)	0.779	0.90 (0.76–1.06)	0.190	1.01 (0.85–1.19)	0.907
Single/unmarried	0.94 (0.75–1.17)	0.557	0.92 (0.73–1.15)	0.462	0.95 (0.77–1.16)	0.588	0.95 (0.77–1.17)	0.620
Unknown	0.81 (0.58–1.12)	0.197	0.77 (0.56–1.07)	0.125	0.85 (0.63–1.15)	0.292	0.82 (0.61–1.11)	0.202
Widowed	1.21 (0.99–1.49)	0.067	1.16 (0.94–1.44)	0.169	1.34 (1.11–1.61)	0.002	1.24 (1.02–1.50)	0.030
Median household income								
< $50,000	Reference				Reference			
$50,000–65,000	1.07 (0.88–1.29)	0.495	1.09 (0.90–1.32)	0.371	1.11 (0.93–1.31)	0.241	1.05(0.89–1.25)	0.549
> $65,000	0.80 (0.71–0.91)	0.001	0.93 (0.770–1.111)	0.405	0.79 (0.70–0.88)	< 0.001	0.87 (0.74–1.03)	0.101
Grade								
I	Reference				Reference			
II	1.22 (0.61–2.45)	0.568	1.19 (0.59–2.39)	0.631	1.10 (0.63–1.91)	0.743	1.09 (0.62–1.90)	0.773
III	1.79 (0.96–3.35)	0.067	1.67 (0.89–3.13)	0.112	1.33 (0.81–2.19)	0.256	1.30 (0.79–2.14)	0.310
IV	1.73 (0.92–3.24)	0.089	1.55 (0.82–2.93)	0.176	1.32 (0.80–2.17)	0.282	1.24 (0.75–2.05)	0.412
Unknown	1.44 (0.76–2.71)	0.259	1.35 (0.71–2.56)	0.358	1.10 (0.66–1.81)	0.724	1.07 (0.64–1.78)	0.797
Histology								
Clear cell	Reference				Reference			
Serous	1.36 (1.14–1.61)	< 0.001	1.28 (1.07–1.53)	0.005	1.25 (1.08–1.45)	0.003	1.23(1.05–1.44)	0.009
Carcinosarcoma	2.07 (1.63–2.61)	< 0.001	2.01 (1.56–2.59)	< 0.001	1.88 (1.52–2.33)	< 0.001	1.87 (1.48–2.37)	< 0.001
TNM stage								
T1N0M0	Reference				Reference			
T2N0M0	2.09 (1.69–2.59)	< 0.001	1.95 (1.57–2.42)	< 0.001	1.85 (1.53–2.23)	< 0.001	1.72 (1.42–2.08)	< 0.001
T3-4aN0MO	3.21 (2.66–3.87)	< 0.001	3.191 (2.63–3.88)	< 0.001	2.70 (2.29–3.19)	< 0.001	2.72 (2.29–3.23)	< 0.001
TanyN1M0	3.68 (3.12–4.33)	< 0.001	3.86 (3.24–4.61)	< 0.001	3.09 (2.68–3.57)	< 0.001	3.41 (2.92–3.99)	< 0.001
TanyN2M0	4.94 (3.65–6.69)	< 0.001	4.92 (3.59–6.74)	< 0.001	3.88 (2.90–5.21)	< 0.001	4.08 (3.01–5.52)	< 0.001
TanyNanyM1	8.82 (7.28–10.67)	< 0.001	8.67 (7.03–10.68)	< 0.001	6.94 (5.82–8.27)	< 0.001	6.94 (5.73–8.41)	< 0.001
Surgery mode								
Total hysterectomy	Reference				Reference			
Extended hysterectomy	1.37 (1.14–1.64)	0.001	1.05 (0.87–1.26)	0.635	1.26 (1.07–1.50)	0.007	0.99 (0.83–1.18)	0.895
Lymphadenectomy								
Yes	Reference				Reference			
No	1.60 (1.37–1.87)	< 0.001	1.34 (1.13–1.58)	< 0.001	1.73 (1.50–1.99)	< 0.001	1.46 (1.25–1.70)	< 0.001
SLN biopsy/removed	0.91 (0.67–1.23)	0.519	0.975 (0.716–1.327)	0.871	0.89 (0.67–1.18)	0.414	0.94 (0.70–1.26)	0.685
Tumor Size (mm)								
< 20	Reference				Reference			
20–39	1.40 (1.10–1.77)	0.006	1.14 (0.89–1.45)	0.291	1.41 (1.13–1.75)	0.002	1.19 (0.96–1.48)	0.122
40–59	2.09 (1.65–2.64)	< 0.001	1.42 (1.12–1.81)	0.003	1.99 (1.61–2.47)	< 0.001	1.43 (1.15–1.78)	0.001
60–79	2.34 (1.80–3.03)	< 0.001	1.34 (1.02–1.74)	0.033	2.35 (1.86–2.98)	< 0.001	1.42 (1.11–1.80)	0.005
≥ 80	3.31 (2.57–4.26)	< 0.001	1.74 (1.34–2.26)	< 0.001	3.29 (2.61–4.15)	< 0.001	1.87 (1.47–2.37)	< 0.001
Unknown	1.62 (1.28–2.04)	< 0.001	1.24 (0.98–1.57)	0.076	1.65 (1.34–2.04)	< 0.001	1.31 (1.06–1.62)	0.014

*RT* radiotherapy, *CRT* chemoradiotherapy, *SLN* sentinel lymph node. Race Others^a^: American Indian, Asian/Pacific Islander. *IPTW* inverse probability treatment weighting

**Table 3:** Survival analysis of predicting CSS and OS after IPTW-adjusted by CRT or CT in NEEC patients

	**Cause-specific survival**				**Overall survival**			
	Univariate analysis		Multivariate analysis		Univariate analysis		Multivariate analysis	
Characteristics	HR (95% CI)	*P*	HR (95% CI)	*P*	HR (95% CI)	*P*	HR (95% CI)	*P*
Adjuvant treatment								
CRT	Reference				Reference			
CT alone	1.96 (1.81–2.12)	** < 0.001**	1.25 (1.14–1.37)	** < 0.001**	1.90 (1.76–2.05)	** < 0.001**	1.27 (1.17–1.38)	** < 0.001**
Age group (years)								
< 50	Reference				Reference			
50–65	0.99 (0.77–1.26)	0.908	1.27 (0.99–1.62)	0.058	0.99 (0.78–1.25)	0.917	1.27 (1.08–1.61)	**0.044**
> 65	1.16 (0.91–1.42)	0.221	1.53 (1.19–1.95)	**0.001**	1.25 (0.99–1.57)	0.062	1.62 (1.28–2.05)	** < 0.001**
Year of diagnosis								
2004–2008	Reference				Reference			
2009–2013	0.78 (0.69–0.86)	** < 0.001**	0.84 (0.76–0.94)	**0.002**	0.80 (0.72–0.88)	** < 0.001**	0.87 (0.78–0.96)	**0.006**
2014–2018	0.69 (0.62–0.76)	** < 0.001**	0.726 (0.649–0.812)	** < 0.001**	0.71 (0.64–0.78)	** < 0.001**	0.75 (0.68–0.84)	** < 0.001**
Race recode								
Black	Reference				Reference			
Others^a^	0.74 (0.63–0.86)	** < 0.001**	0.68 (0.58–0.80)	** < 0.001**	0.76 (0.66–0.88)	** < 0.001**	0.72 (0.62–0.83)	** < 0.001**
White	0.87 (0.79–0.96)	**0.003**	0.85 (0.77–0.93)	**0.001**	0.87 (0.79–0.95)	**0.001**	0.85 (0.80–0.93)	** < 0.001**
Marital status								
Divorced/separated	Reference				Reference			
Married	0.88 (0.78–0.98)	**0.026**	0.95 (0.85–1.07)	0.438	0.85 (0.76–0.95)	**0.005**	0.93 (0.8301.04)	0.199
Single/unmarried	1.09 (0.88–1.16)	0.915	0.97 (0.84–1.12)	0.655	0.98 (0.86–1.12)	0.794	0.96 (0.84–1.09)	0.502
Unknown	0.76 (0.61–0.96)	**0.018**	0.89 (0.71–1.12)	0.310	0.81 (0.65–0.99)	**0.041**	0.93 (0.76–1.15)	0.516
Widowed	1.13 (0.98–1.29)	0.095	1.018 (0.883–1.172)	0.809	1.18 (1.04–1.34)	**0.012**	1.06 (0.93–1.21)	0.413
Median household income								
< $50,000	Reference				Reference			
$50,000–65,000	1.01 (0.89–1.16)	0.908	1.11 (0.98–1.26)	0.115	0.98(0.87–1.11)	0.738	1.07 (0.95–1.21)	0.257
> $65,000	0.90 (0.80–1.01)	0.077	0.99 (0.88–1.12)	0.887	0.85(0.76–0.96)	**0.006**	0.94 (0.84–1.05)	0.270
Grade								
I	Reference				Reference			
II	1.14 (0.70–1.86)	0.588	1.10 (0.68–1.79)	0.698	1.07 (0.70–1.64)	0.750	1.05 (0.69–1.61)	0.814
III	1.68 (1.09–2.58)	**0.019**	1.42 (0.92–2.19)	0.115	1.40 (0.96–2.04)	0.076	1.20 (0.83–1.76)	0.333
IV	1.65 (1.07–2.55)	**0.023**	1.38 (0.89–2.13)	0.149	1.41 (0.97–2.06)	0.073	1.20 (0.82–1.75)	0.354
Unknown	1.52 (0.98–2.34)	0.062	1.22 (0.79–1.89)	0.381	1.31 (0.90–1.92)	0.160	1.07 (0.73–1.57)	0.715
Histology								
Clear cell	Reference				Reference			
Serous	1.12 (0.99–1.27)	0.083	1.07 (0.94–1.21)	0.322	1.08 (0.96–1.21)	0.225	1.03 (0.92–1.16)	0.610
Carcinosarcoma	1.73 (1.47–2.03)	** < 0.001**	1.90 (1.597–2.252)	** < 0.001**	1.67 (1.43–1.94)	** < 0.001**	1.81 (1.54–2.13)	** < 0.001**
TNM stage								
T1N0M0	Reference				Reference			
T2N0M0	2.20 (1.78–2.71)	** < 0.001**	2.11 (1.71–2.61)	** < 0.001**	2.09 (1.73–2.537)	** < 0.001**	2.02 (1.67–2.44)	** < 0.001**
T3-4aN0MO	3.74 (3.20–4.36)	** < 0.001**	3.43 (2.933–4.013)	** < 0.001**	3.30 (2.87–3.79)	** < 0.001**	3.05 (2.64–3.52)	** < 0.001**
TanyN1M0	4.22 (3.67–4.86)	** < 0.001**	4.12 (3.566–4.766)	** < 0.001**	3.86 (3.40–4.38)	** < 0.001**	3.78 (3.32–4.31)	** < 0.001**
TanyN2M0	5.58 (4.40–7.08)	** < 0.001**	5.21 (4.082–6.648)	** < 0.001**	4.78 (3.80–6.00)	** < 0.001**	4.43 (3.51–5.60)	** < 0.001**
TanyNanyM1	10.62(9.32–12.10)	** < 0.001**	8.02 (6.964–9.235)	** < 0.001**	9.06 (8.05–10.20)	** < 0.001**	6.85 (6.03–7.79)	** < 0.001**
Surgery mode								
Total hysterectomy	Reference				Reference			
Extended hysterectomy	1.53 (1.37–1.71)	** < 0.001**	1.14 (1.02–1.28)	**0.021**	1.49 (1.34–1.66)	** < 0.001**	1.13 (1.06–1.26)	**0.026**
Lymphadenectomy								
Yes	Reference				Reference			
No	2.33 (2.15–2.53)	** < 0.001**	1.47 (1.34–1.61)	** < 0.001**	2.24 (2.07–2.42)	** < 0.001**	1.45 (1.32–1.58)	** < 0.001**
SLN biopsy/removed	0.86 (0.69–1.07)	0.182	1.02 (0.81–1.28)	0.883	0.85 (0.70–1.05)	0.137	0.99 (0.80–1.24)	0.970
Tumor Size (mm)								
< 20	Reference				Reference			
20–39	1.30 (1.11–1.53)	**0.001**	1.08 (0.92–1.26)	0.378	1.31 (1.13–1.53)	** < 0.001**	1.10 (0.94–1.28)	0.241
40–59	1.72 (1.47–2.02)	** < 0.001**	1.26 (1.07–1.48)	**0.006**	1.70 (1.46–1.97)	** < 0.001**	1.24 (1.07–1.45)	**0.005**
60–79	2.21 (1.87–2.63)	** < 0.001**	1.27 (1.07–1.52)	**0.007**	2.22 (1.89–2.61)	** < 0.001**	1.29(1.10–1.53)	**0.002**
≥ 80	2.80 (2.37–3.32)	** < 0.001**	1.39 (1.17–1.66)	** < 0.001**	2.80 (2.39–3.29)	** < 0.001**	1.43 (1.21–1.69)	** < 0.001**
Unknown	1.82 (1.56–2.11)	** < 0.001**	1.18 (1.01–1.37)	**0.041**	1.82 (1.58–2.10)	** < 0.001**	1.21 (1.05–1.40)	**0.010**

*CT* chemotherapy, *CRT* chemoradiotherapy, *SLN* sentinel lymph node. Race Others^a^: American Indian, Asian/Pacific Islander. *IPTW* inverse probability treatment weighting

### Exploratory Subgroup Analysis in stage I-IV NEEC patients

**Fig. 1:**
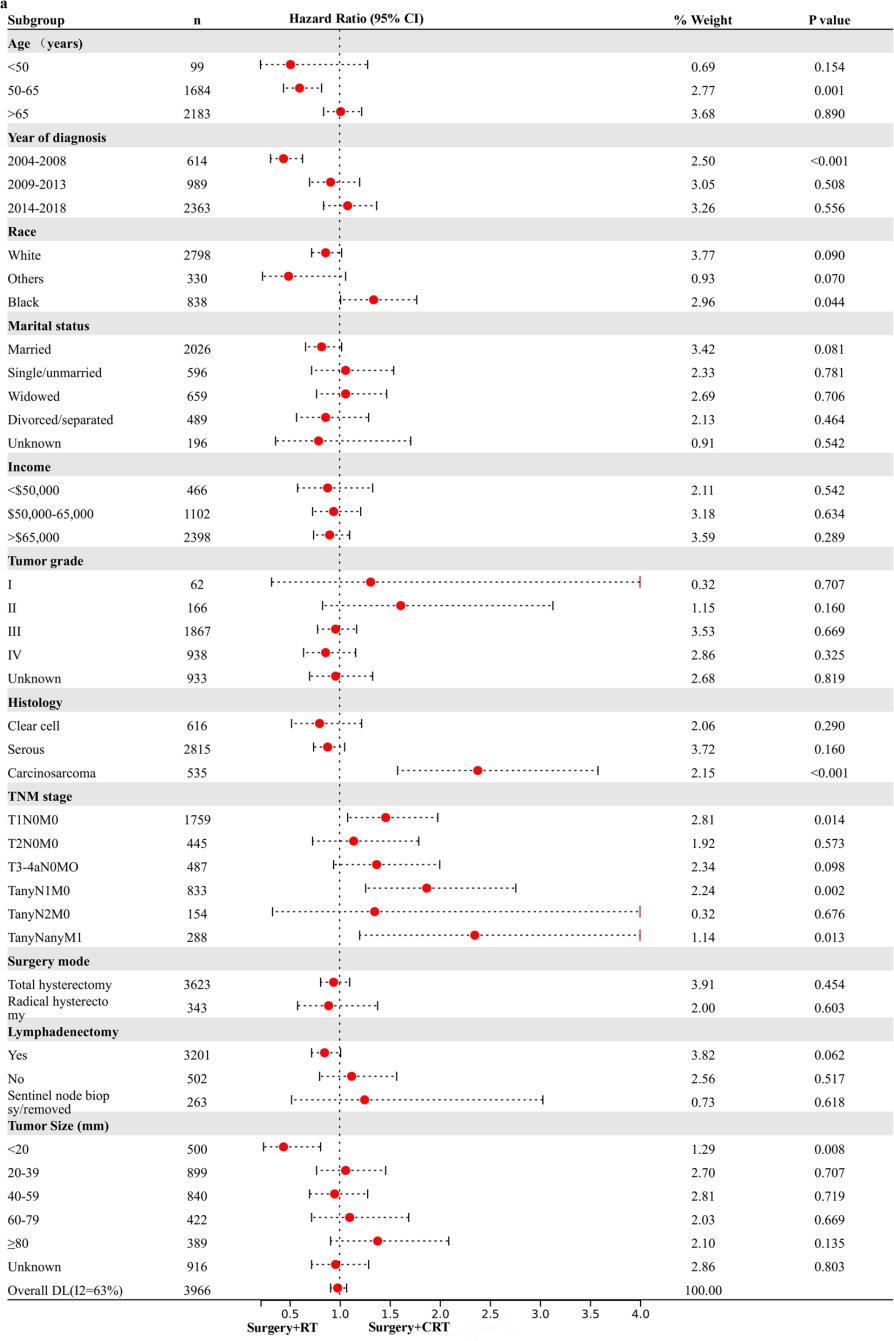

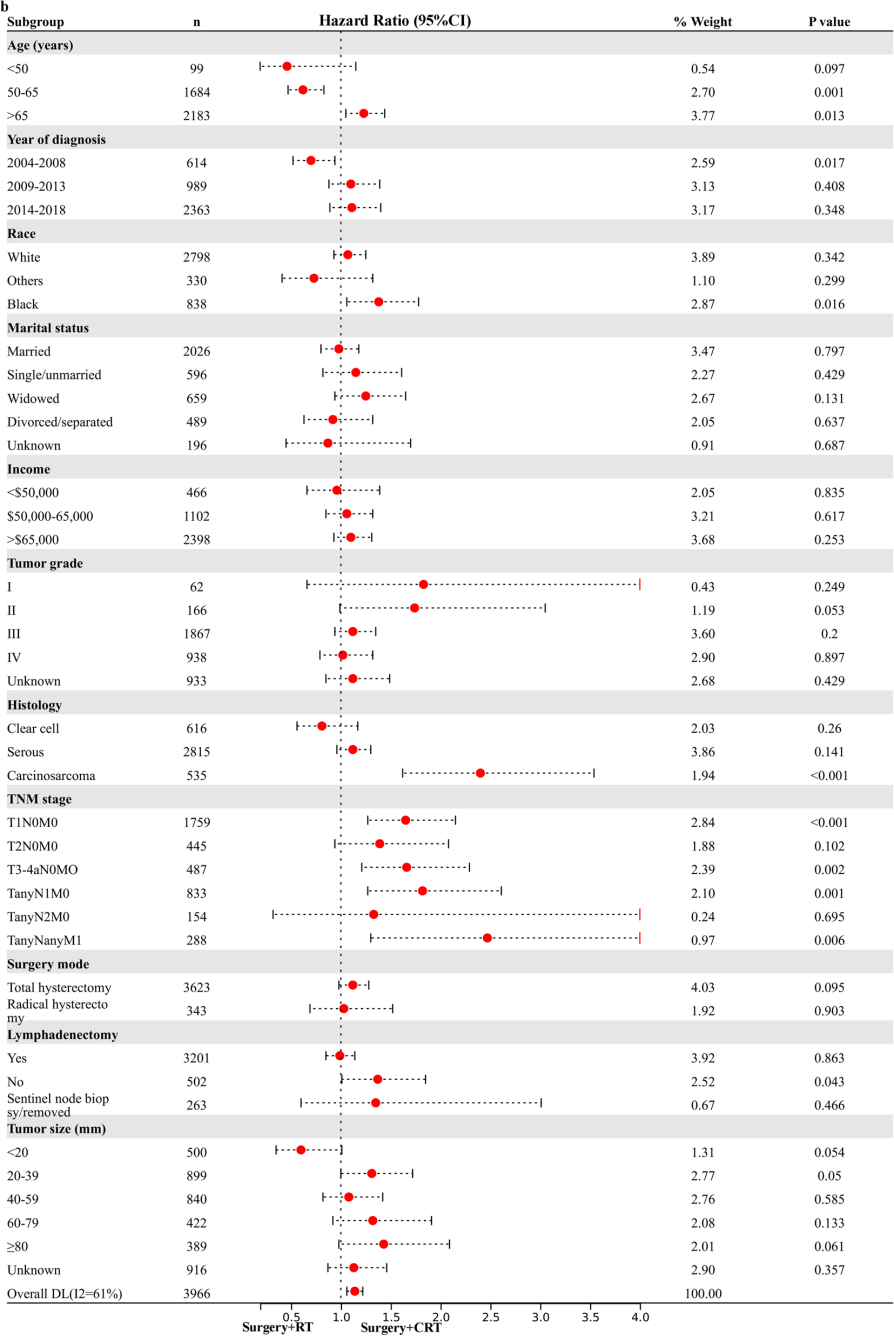

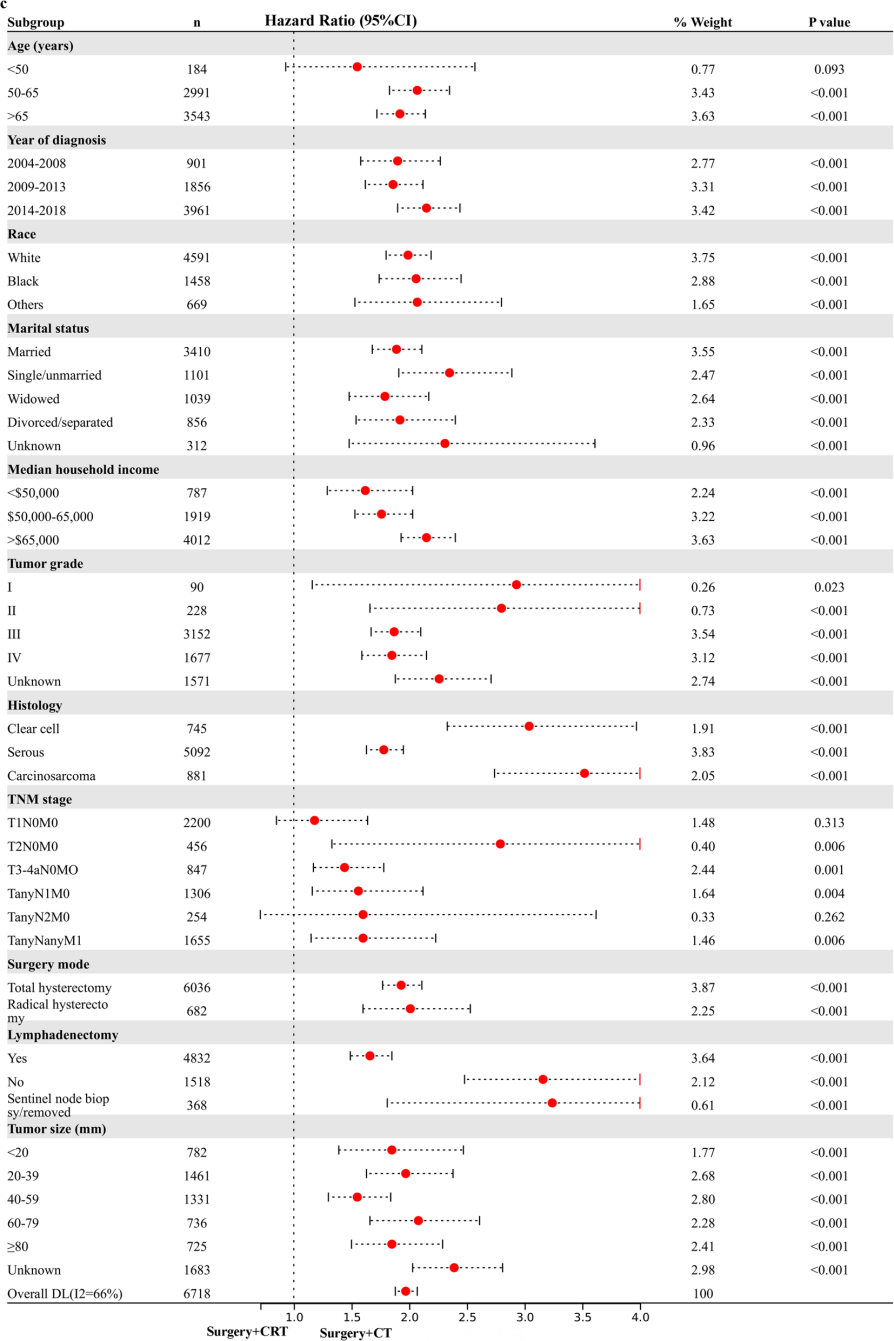

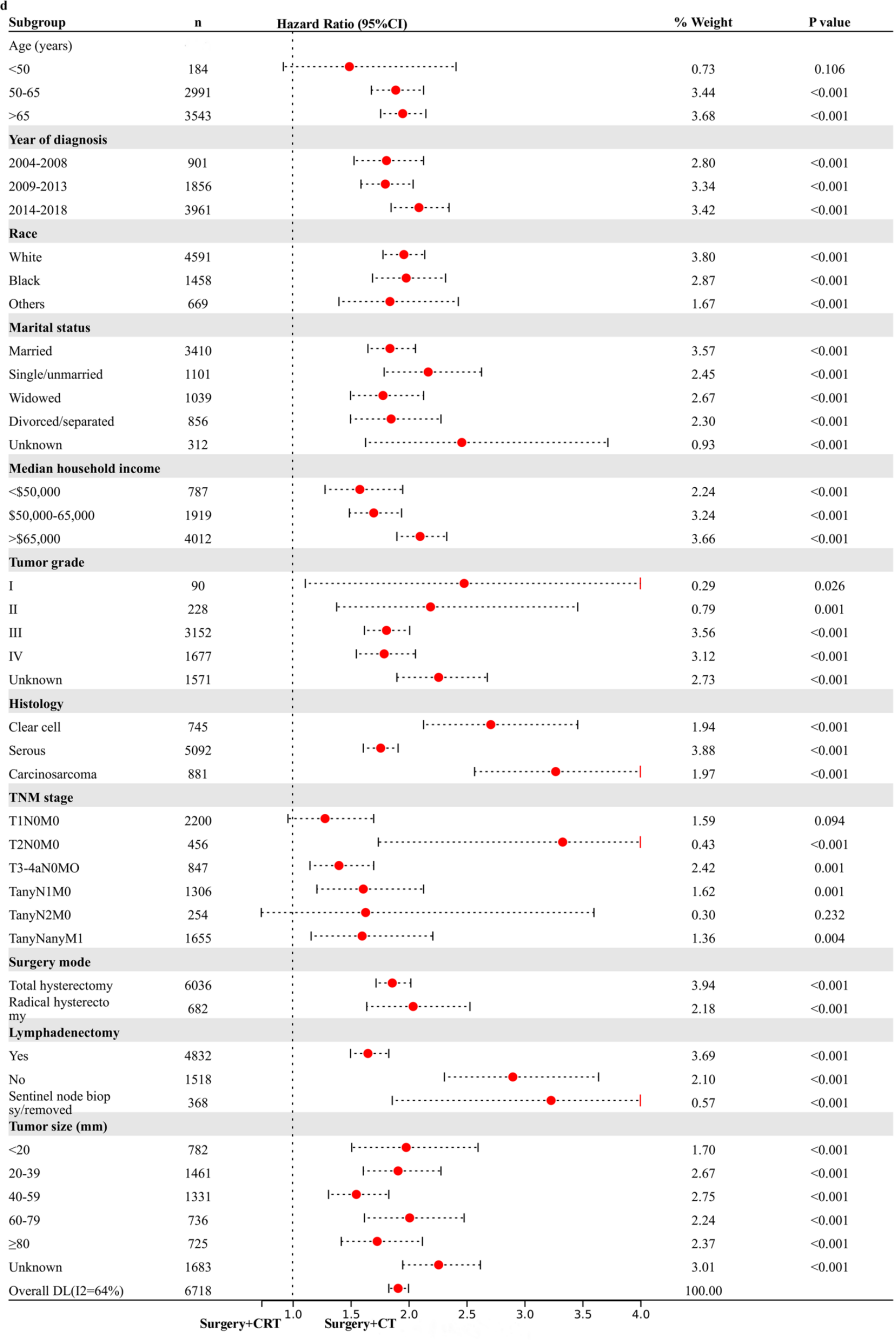
Exploratory subgroup analysis concerning postoperative adjuvant treatment impact on survival outcome in the whole cohort. **a** Cause-specific survival after IPTW-adjustment stratified by CRT and RT. **b** Overall survival after IPTW-adjustment stratified by CRT and RT. **c** Cause-specific survival after IPTW-adjustment stratified by CRT and CT. **d** Overall survival after IPTW-adjustment stratified by CRT and CT. CI: confidence interval; HR: hazard ratio; IPTW: inverse probability of treatment weighting; CRT: chemoradiotherapy; CT: chemotherapy; RT: radiotherapy. The vertical solid-line refers to a hazard ratio of 1.0. *P* < 0.05 indicates statistical significance

Based on the above analysis, CRT showed beneficial effect of survival outcome compared to CT alone, however, similar impact to RT alone in UVA analysis. The result promoted us to further explore who will finally benefit from the combined CRT treatment. An exploratory subgroup analysis was conducted in selected subgroups related to prognosis, as shown in the forest plot (Fig. 1). Before and after matching, heterogeneity was high (I2 > 50%) on fixed-effects model, therefore, we employed the random-effects model to illustrate the result. After IPTW-adjustment, most subgroups showed similar survival outcome between RT alone and CRT given. Interestingly, possible improved CSS (Fig. 1a) and OS (Fig. 1b) were observed after CRT administration in various TNM stage subgroups and histology of carcinosarcoma. No survival difference was demonstrated in serous and clear cell subgroups for RT alone compared to CRT administered. When compared to CT alone, CRT given posed beneficial impact on CSS (Fig. 1c) and OC (Fig. 1d) in most subgroups with statistical significance (*p* < 0.05), verifying addition of RT to CT benefits most patients.

### Sensitivity analysis among various TNM stages in serous and clear cell histology

**Fig. 2:**
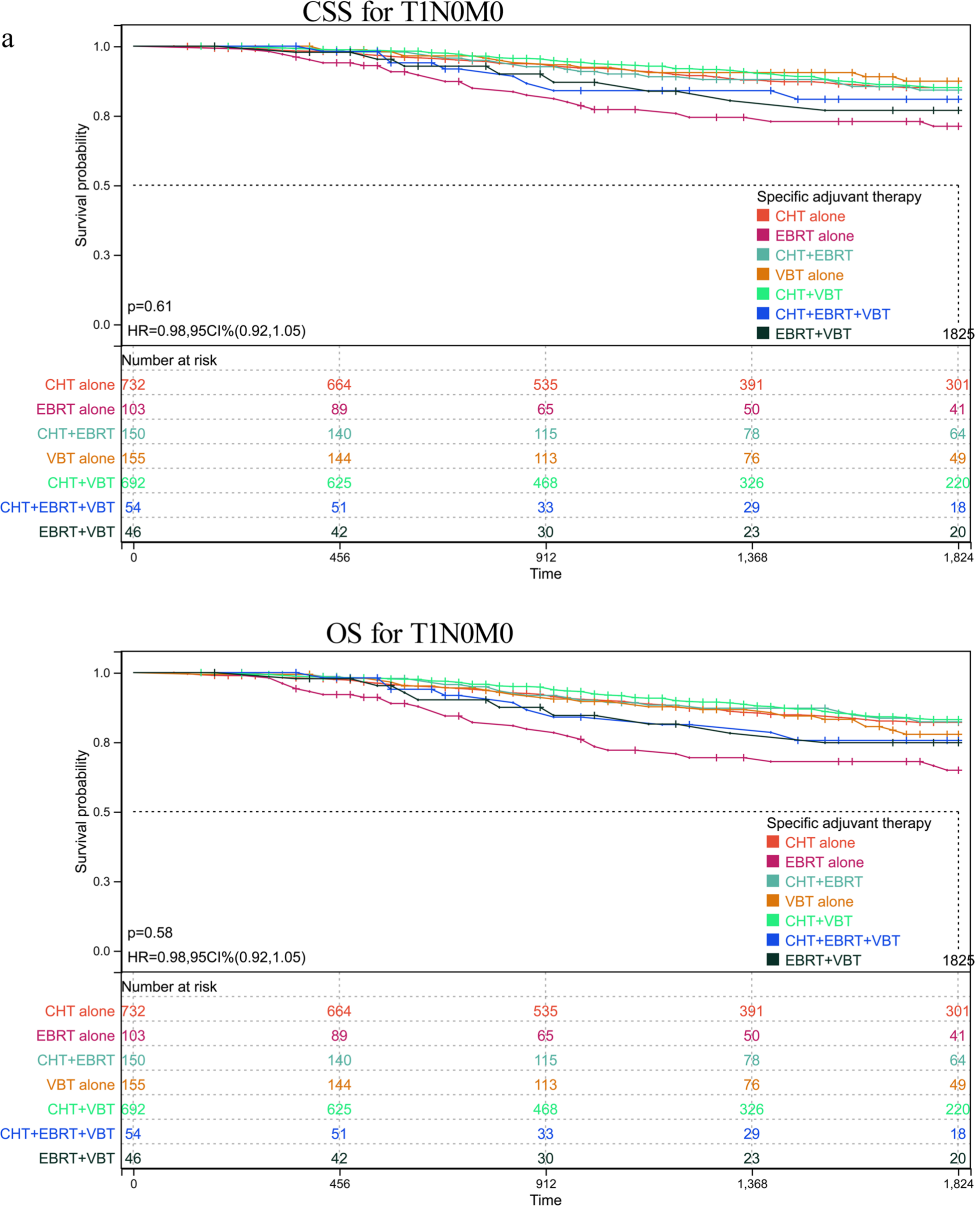

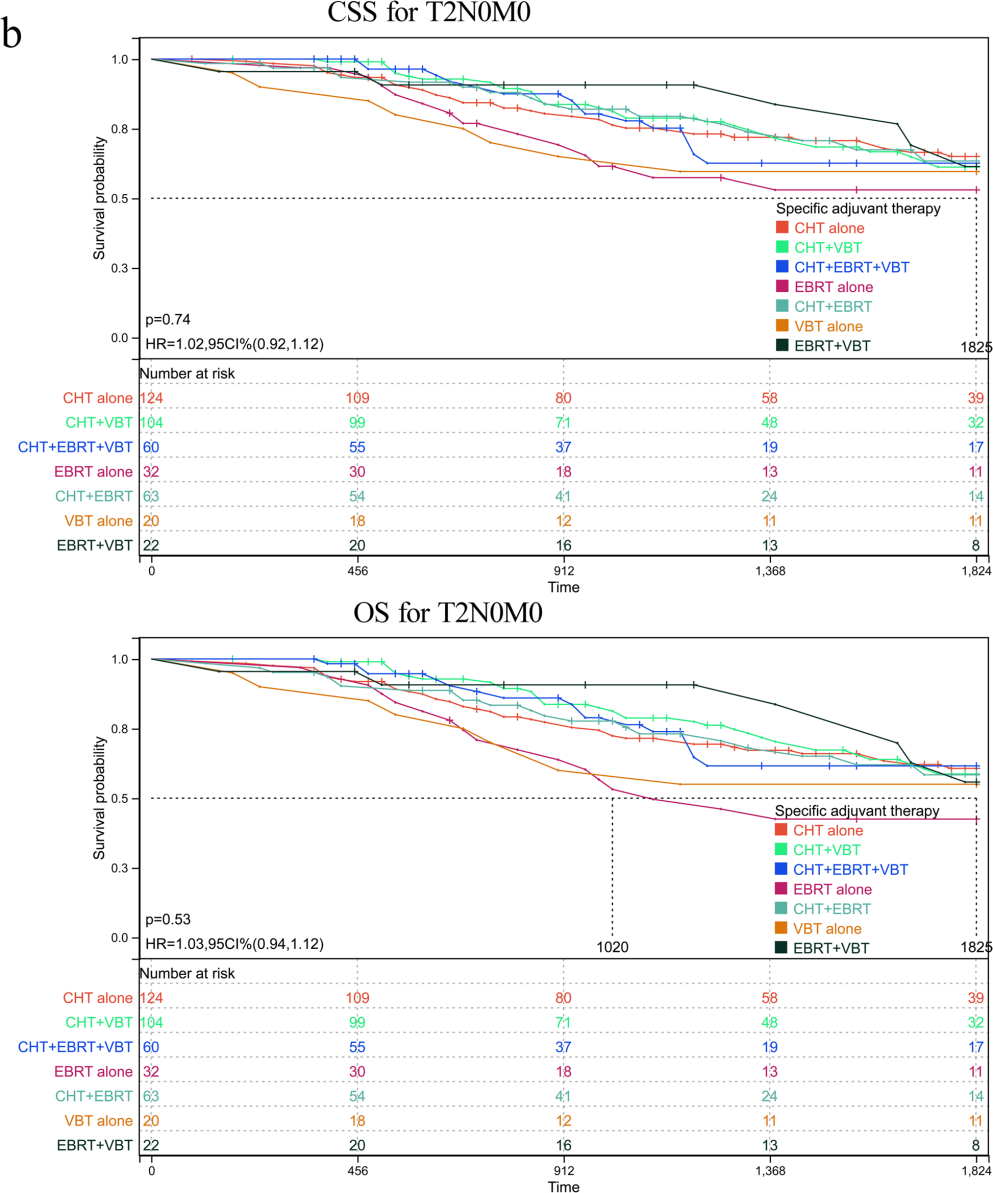

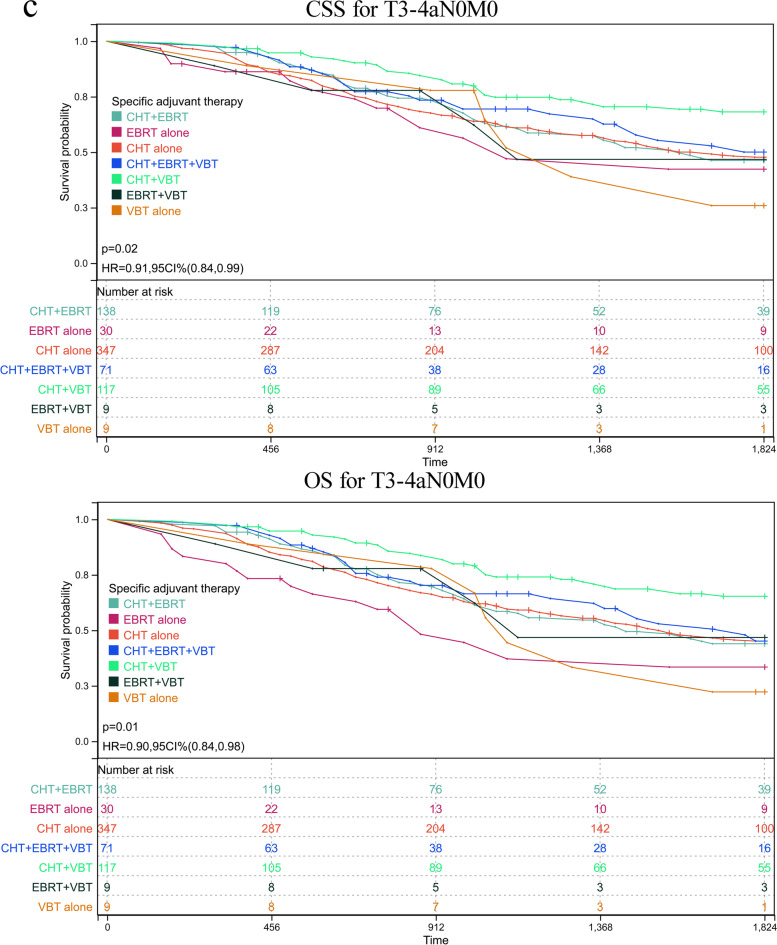

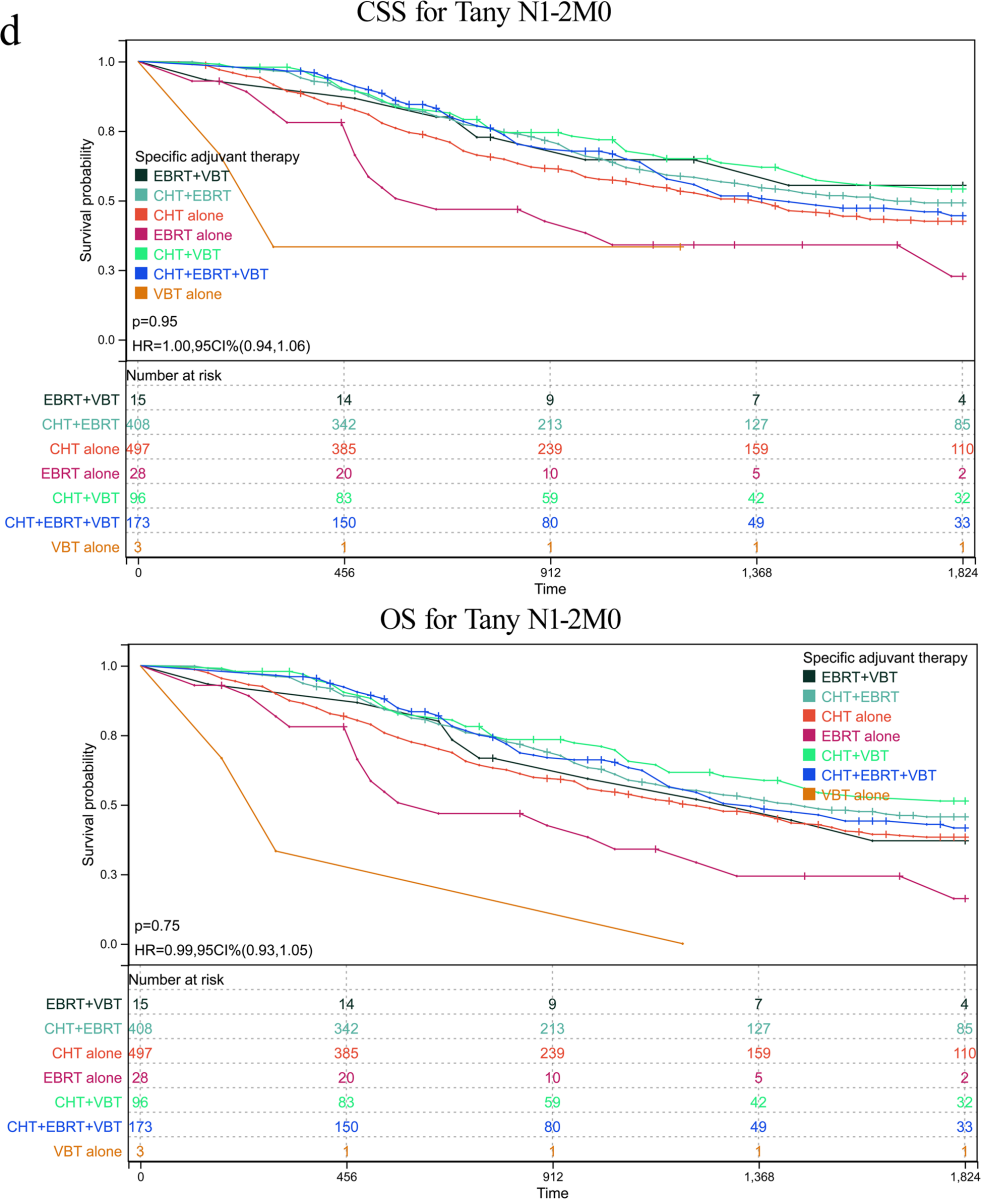

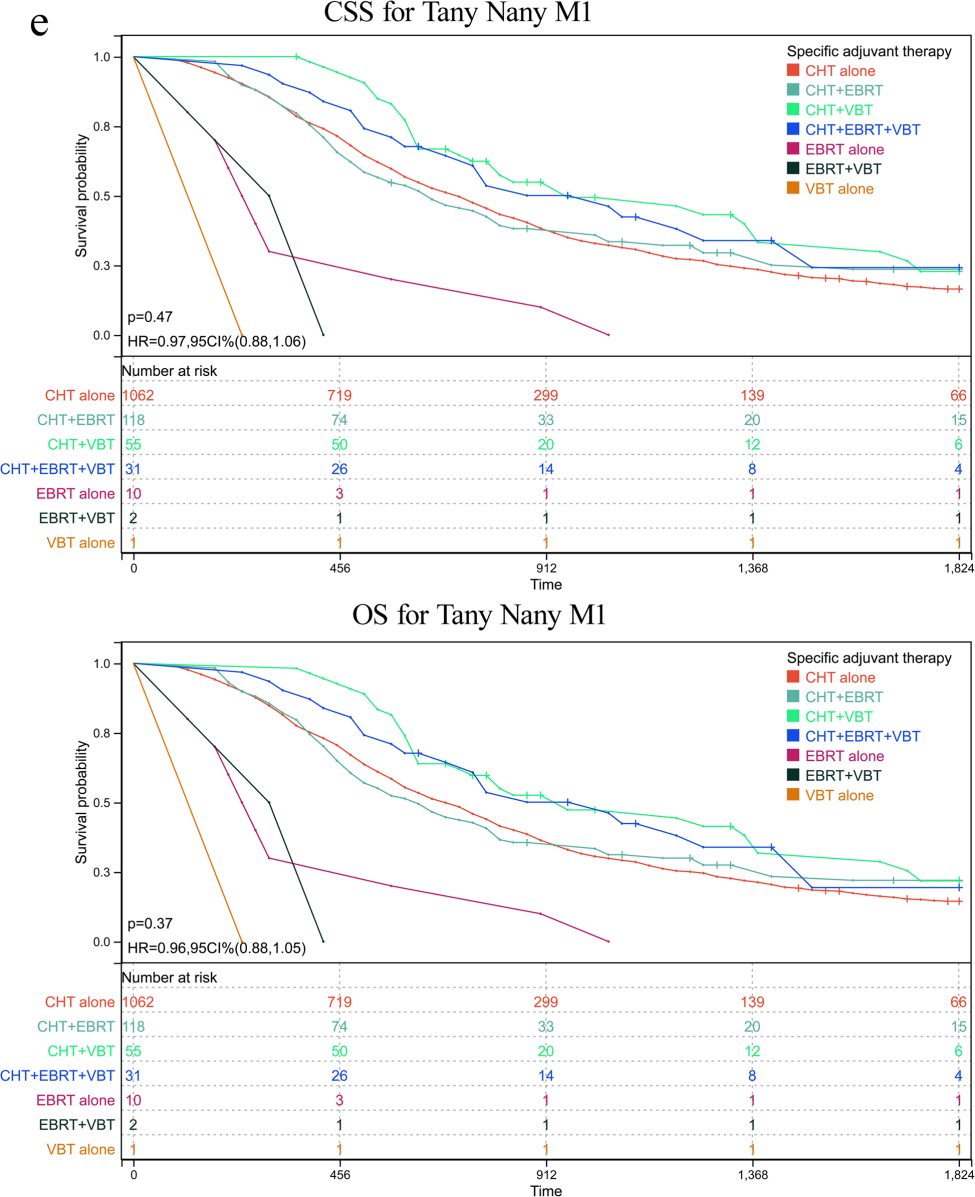
Sensitivity analyses for various treatment modalities on patients with serous histology. **a** CSS and OS in T1N0M0. **b** CSS and OS in T2N0M0. **c** CSS and OS in T3-4aN0M0. **d** CSS and OS in TanyN1-2M0. **e** CSS and OS in TanyNanyM1. CI: confidence interval; HR: hazard ratio; CSS: cause-specific survival; OS: overall survival; VBT: vaginal brachytherapy; EBRT: external beam radiotherapy; CT: chemotherapy. *P* < 0.05 indicates statistical significance

As abovementioned, patients with serous histology represented 74.23% (5577/7513) of the whole cohort, among of which 25.55% of participants in this subgroup had reached at least 5 years of follow-up. To further validate the role of various adjuvant modalities in treating patients with serous histology, we conducted a sensitivity analysis by comparing the OS and CSS based on TNM stages. Patients were classified based on whether or not they received any RT or CT and on the number receiving specific RT type. The adjuvant therapy was classified as follows: VBT alone, EBRT alone, CT alone, VBT + EBRT, VBT + CT, EBRT + CT, VBT + EBRT + CT. Estimated CSS and OS adjusted for stratification factors at 5 years was concluded in Table 4 and Fig. 2. In T1N0M0 (Fig. 2a) and T2N0M0 (Fig. 2b), VBT alone showed similar CSS and OS impact to other modalities, with the exception of EBRT alone in T1N0M0 stage, with 5-year CSS 62.81% in EBRT plus CT versus 52.41% in EBRT alone group and corresponding OS 57.96% versus 42.36%. In T3-4aN0M0 (Fig. 2c), a survival improvement was observed for CSS and OS after performance of VBT combined with CT. When pelvic or para-aortic nodal metastasis (TanyN1-2M0) was identified, combination of CT with any method of RT posed beneficial effect than EBRT or CT alone (Fig. 2d). Whereas distant metastasis was confirmed (TanyNanyM1), combination of CT with EBRT or VBT was beneficial than CT alone, although CT alone was more given than CRT (Fig. 2e). However, most of UCS patients died or lost to follow-up within five years after initial diagnosis, hindering 5-year survival analysis. For CCC patients, similar 5-year survival rate to that of SC patients was observed in T1N0M0 stage, and better survival compared to that of SC cases in advanced stage. However, limited number of CCC patients affected the final survival analysis separated by tumor stage and addition therapies, which may explain no significant difference among various adjuvant treatments (supplementary table 2).

**Table 4:** Estimated five-year OS and CSS in endometrial serous carcinoma stratified by TNM stages and adjuvant treatments

		**Cause-specific survival**			**Overall survival**		
**TNM stage**	**Number**	**Estimate, % (95%CI)**	**Hazard ratio (95%CI)**	***P*** ** value**	**Estimate, % (95%CI)**	**Hazard ratio (95%CI)**	***P*** ** value**
T1N0M0	1932	84.64%(82.67–86.41)			81.41%(79.32–83.32)		
EBRT + CT	150	84.17% (75.77–89.86)	Reference		82.24% (73.53–88.31)	Reference	
CT alone	732	84.96% (81.59–87.76)	1.037 (0.620–1.734)	0.889	81.93% (78.38–84.96)	1.131 (0.698–1.835)	0.617
EBRT alone	103	70.84% (59.45–79.56)	2.281 (1.220–4.264)	**0.010**	64.96% (53.59–74.20)	2.507 (1.406–4.470)	**0.002**
VBT alone	155	87.19% (78.87–92.39)	0.921 (0.453–1.872)	0.820	77.76% (68.15–84.79)	1.457 (0.796–2.666)	0.223
EBRT + VBT	46	76.67% (58.17–87.79)	1.257 (0.540–2.922)	0.596	74.73% (56.51–86.18)	1.256 (0.567–2.782)	0.574
VBT + CT	692	85.04% (81.12–88.21)	0.999 (0.588–1.699)	0.998	82.93% (78.92–86.24)	1.068 (0.648–1.760)	0.796
EBRT + VBT + CT	54	79.93% (63.26–89.62)	1.189 (0.514–2.753)	0.686	74.60% (57.47–85.64)	1.384 (0.644–2.974)	0.405
T2N0M0	425	63.82%(58.47–68.68)			59.37%(54.02–64.31)		
EBRT + CT	63	62.81% (45.27–76.11)	Reference		57.96% (41.51–71.30)	Reference	
CT alone	124	65.01% (54.33–73.78)	1.053 (0.582–1.902)	0.865	60.71% (50.26–69.62)	0.987 (0.578–1.685)	0.961
EBRT alone	32	52.41% (32.10–69.25)	1.648 (0.792–3.426)	0.181	42.36% (24.46–59.22)	1.730 (0.906–3.303)	0.097
VBT alone	20	59.27% (34.74–77.20)	1.395 (0.596–3.262)	0.443	55.00% (31.34–73.49)	1.260 (0.573–2.771)	0.565
EBRT + VBT	22	62.15% (33.39–81.37)	0.927 (0.363–2.370)	0.874	56.06% (28.55–76.55)	0.865 (0.366–2.047)	0.741
VBT + CT	104	61.36% (49.08–71.52)	1.032 (0.562–1.893)	0.919	58.70% (46.43–69.07)	0.882 (0.504–1.542)	0.659
EBRT + VBT + CT	60	63.63% (46.53–76.56)	1.047 (0.518–2.119)	0.897	62.34% (45.51–75.30)	0.894 (0.463–1.725)	0.738
T3-4aN0M0	721	51.77% (47.77–55.61)			48.43%(44.49–52.25)		
EBRT + CT	138	47.04% (37.16–56.30)			44.45% (34.91–53.55)		
CT alone	347	47.55% (41.57–53.28)	0.945 (0.696–1.284)	0.719	45.05% (39.20–50.72)	0.936 (0.695–1.261)	0.664
EBRT alone	30	45.28% (22.55–61.30)	0.847 (0.463–1.547)	0.588	33.77% (17.33–51.05)	0.985 (0.573–1.692)	0.956
VBT alone	9	23.47% (2.64–56.10)	1.340 (0.573–3.132)	0.499	20.00% (2.30–50.31)	1.456 (0.654–3.242)	0.357
EBRT + VBT	9	47.27% (12.00–76.74)	0.578 (0.202–1.651)	0.306	47.27% (12.00–76.74)	0.504 (0.173–1.468)	0.209
VBT + CT	117	68.24% (58.23–76.33)	0.595 (0.386–0.916)	**0.018**	65.50% (55.41–73.83)	0.584 (0.385–0.888)	**0.012**
EBRT + VBT + CT	71	49.20% (34.22–62.55)	0.908 (0.572–1.441)	0.681	44.84% (30.50–58.17)	0.966 (0.621–1.503)	0.879
TanyN1-2M0	1220	47.64%(44.52–50.69)			43.64%(40.60–46.64)		
EBRT + CT	408	49.13% (43.08–54.88)			45.51% (39.65–51.18)		
CT alone	497	42.32% (37.23–47.31)	1.239 (1.012–1.518)	**0.038**	38.06% (33.19–42.91)	1.249 (1.029–1.514)	**0.024**
EBRT alone	28	26.43% (9.27–47.49)	1.945 (1.180–3.205)	**0.009**	19.38% (6.00–38.41)	2.045 (1.273–3.286)	**0.003**
VBT alone	3		3.132 (0.767–12.792)	0.112		4.391 (1.385–13.920)	0.012
EBRT + VBT	15	55.78% (26.35–77.45)	0.958 (0.413–2.222)	0.920	36.43% (13.18–60.45)	1.279 (0.631–2.592)	0.496
VBT + CT	96	53.94% (42.02–64.44)	0.863 (0.600–1.242)	0.429	51.19% (39.54–61.67)	0.851 (0.601–1.205)	0.364
EBRT + VBT + CT	173	43.99% (34.38–53.17)	1.050 (0.789–1.396)	0.740	41.17% (31.82–50.26)	1.026 (0.779–1.350)	0.857
TanyNanyM1	1279	21.45% (19.11–23.89)			18.92%(16.72–21.24)		
EBRT + CT	118	23.63% (15.35–32.93)	Reference		21.88% (14.10–30.76)		
CT alone	1062	16.48% (13.75–19.42)	0.954 (0.752–1.208)	0.694	14.51% (12.02–17.22)	0.957 (0.760–1.206)	0.710
EBRT alone	10	0	3.700 (1.901–7.199)	< 0.001	0	3.538 (1.822–6.871)	< 0.001
VBT alone	1	0	7.639 (1.045–55.816)	0.045	0	7.644 (1.047–55.789)	0.045
EBRT + VBT	2	0	4.994 (1.215–20.518)	0.026	0	4.853 (1.183–19.914)	0.028
VBT + CT	55	22.81% (10.45–38.02)	0.656 (0.433–0.992)	0.046	21.83% (10.00–36.58)	0.657 (0.439–0.982)	0.041
EBRT + VBT + CT	31	23.13% (8.84–41.33)	0.721 (0.444–1.170)	0.185	19.09% (6.46–36.76)	0.718 (0.447–1.152)	0.170

*CT* chemotherapy, *EBRT* external beam radiotherapy, *VBT* vaginal brachytherapy

## Discussion

The current study was a retrospective population-based analysis with, to our knowledge, the largest sample size for exclusive non-endometrioid endometrial carcinomas which encompassed a relatively complete spectrum of histology. Through SEER database exploration, we demonstrated important prognostic factors affecting CSS and OS, including patients’ age, cancer histology, tumor size, TNM stage and adjuvant treatment options. Particular attention was paid to the survival benefit of adjuvant modalities for NEEC patients who underwent TAH-based surgery. As a result, vaginal brachytherapy plus chemotherapy deserved the most beneficial effect than any other single or combined options. Sensitivity analysis was conducted to assess the reliability of results in patients with serous histology, proving the estimated effect in the primary analysis.

In the current study, histology was the most important factor affecting CSS and OS. Patients with UCS showed a significantly shorter survival period than SC and CCC, both before and after IPTW-adjustment by adjuvant treatment, which was in agreement with a recent large cohort meta-analysis that reported UCS with an increased risk of death compared to SC and CCC [16]. Prior reports have indicated that serous cancer was more chemo-responsive than clear cell subtype [17], therefore, separate evaluations in terms of various adjuvant modalities impact on uterine SC and CCC are necessary. Fortunately, our study’s large number of serous carcinoma cases allowed further analysis of the various adjuvant treatments stratified by stage.

For women with early-stage SC, this analysis indicated changing trend in terms of adjuvant therapy, specifically speaking, adjuvant CT and VBT were more administered compared to EBRT. In addition, similar benefits of VBT alone were shown compared to other adjuvant options, with the exception of EBRT alone which showed worst benefit. However, addition of CT to VBT did not show survival benefit. These results agreed with the report from the National Cancer Database (NCDB) in which adjuvant CT and VBT have been increasingly administered, whereas the use of EBRT has decreased [18]. The trend from EBRT to VBT may be partially attributed to the PORTEC-2 trial which analyzed high-intermediate risk endometrial cancer and found VBT to be non-inferior and resulted in fewer side effects compared to EBRT [19]. Accordingly, some current guidelines recommend adjuvant VBT for women with early stage high-intermediate risk endometrial cancer [20]. However, conflicting results persisted with respect to VBT as a complete substitute of EBRT in patients with stage I USC. The current analysis and existing literature revealed VBT alone might be an option in early stage SC, while the combined schedule should mainly be recommended for women in advanced stage. For patients in stage III-IVA without nodal metastasis, better survival outcome was prominent in the combined utilization of CT and VBT, although addition of EBRT to CT did not show survival benefit than CT alone. When positive pelvic or para-aortic nodes were identified, CT plus either EBRT or VBT deserved beneficial impact on survival compared to any single option. This result agreed with one large cohort of NCDB analysis, which indicated the addition of RT to CT promoted survival among women with node-positive serous endometrial cancer [21]. Several retrospective multicenter studies reported a significant benefit of CRT compared with CT or EBRT alone in stage IIIC endometrial cancer [22] or stage III-IV endometrial serous cancer [23]. Conversely, in the randomized GOG-258 trial [10] for stage III-IV endometrial cancer, CRT did not improve OS or PFS, but the rate of pelvic and para-aortic nodal relapse was substantially lower in the CRT arm. With respect to stage IVB or distant metastasis of serous cancer, we found CT with either form of RT prolonged survival than CT alone, although with limited statistical significance given big difference in sample size. As one previous report from Viswanathan and colleagues, women with stage IVB endometrial SC were treated with adjuvant CT and EBRT, appearing decreased rates of tumor recurrence or progression [24].

In the last decade, TCGA research network proposed molecular classification for endometrial cancer to four risk groups: polymerase epsilon exonuclease domain mutated (POLE EDM), mismatch repair deficient (MMRd), p53 wild-type/copy-number- low (p53 wt) and p53-mutated/copy number-high (p53 abn). The molecular characterization changed the traditional risk stratification according to tumor grade and histotype, depth of myometrial invasion and surrounding organ involvement [25]. For patients with NEEC, exploring other mutations in possibly target pathways, such as in FBXW7-FGFR2 or PI3K-AKT pathways, is especially meaningful [26]. In the era of personalized medicine, efforts are persistently required to explore the best strategy for each patient’s profile. More recently, artificial intelligence, especially radiomic profiling, has attracted great attention due to its extraction of mineable high-dimensional data from clinical radiological images, thus providing noteworthy information [27]. Even these advancements as described above, the determination to conduct molecular investigation and employ novel techniques depends on the local resources and arrangements of each center’s multidisciplinary team. Thus, traditional clinicopathological prognostic factors are still considered in clinical routine practice to tailor the personalization of patient therapy.

Although we attempted to account for nonrandom selection of patients, we recognized several inherent methodological limitations. Five questions need to be addressed in the future study. First, the selection bias of retrospective study design represented the main weaknesses of the current study. Our findings remained primarily hypothesis-generating and must be evaluated in the context of randomized evidence, when available. Second, our data lacked detailed information regarding tumor marginal status and lymphovascular invasion (LVSI). However, traditional risk factors in predicting prognosis may not be applicable to high-risk histology, and LVSI was also not prognostic of overall survival according to PORTEC-3 [8]. Third, the database did not contain data regarding RT details (fields, dose, and fractionation) or the effect of course as well as regimen of chemotherapy. Fourth, our analysis focused primarily on OS and CSS, without details concerning local recurrence and distant metastasis after initial treatment due to the unavailability in SEER database, which could have important implications for the impact of adjuvant therapy in this patient population. Lastly, we anticipated that the further adoption of molecular/genomic profiling of NEEC patients might overcome the necessity of exploring combination of various adjuvant strategies.

## Conclusion

The current database analysis included a wide spectrum of NEEC and indicated UCS with a worse prognosis than SC and CCC, justifying a more aggressive adjuvant treatment of combined CT and RT. Given the large sample size of endometrial serous cancer in the whole cohort, insight into the optimal adjuvant management was analyzed based on stage. CT alone still formed the basis of adjuvant treatment, although there is a growing trend to use VBT combined with CT as the adjuvant modality for both early and advanced serous cancer. Both CT and VBT, single or combined, appeared to benefit stage I-II patients with serous histology. In stage III-IV SC patients, CT plus VBT was still associated with improved cancer outcomes. When nodal metastases were identified, addition EBRT to CT may be recommended. More research, ideally in a randomized fashion and even further innovative treatment modalities, is warranted to confirm these results and improve the outcomes for these aggressive tumors.

## Supplementary Information

**Additional file 1: Supplementary Figure 1.** Eligibility, inclusion, and exclusion criteria of the study population.

**Additional file 2: Supplementary Table 1**. Baseline characteristics before and after IPTW-adjusted by postoperative adjuvant treatment. **Supplementary Table 2.** Estimated five-year OS and CSS in endometrial clear cell carcinoma stratified by TNM stages and adjuvant treatments.

## Data Availability

Data from the SEER program is available for public. The data supporting the conclusions of this article are available in the Surveillance Epidemiology, and End Results (SEER) database (https://seer.cancer.gov/).

## Abbreviations

NEEC: Non-endometrioid endometrial carcinomas
SC: Serous carcinoma
UCS: Uterine carcinosarcomas
SEER: Surveillance, Epidemiology, and End Results Program
CCC: Clear cell carcinoma
PSM: Propensity score matching
IPTW: Inverse probability treatment weighting
OS: Overall survival
CSS: Cause-specific survival
CRT: Chemotherapy and radiotherapy
VBT: Vaginal brachytherapy
EBRT: External beam radiotherapy
RT: Radiotherapy
CT: Chemotherapy

## References

[1] Lu KH, Broaddus RR (2020). Endometrial cancer. N Engl J Med.

[2] Yuce Sari S, Guler OC, Oymak E, Gultekin M, Yigit E, Kahvecioglu A (2022). Uterine papillary serous and clear cell carcinomas: comparison of characteristics and clinical outcomes. J Obstet Gynaecol Res.

[3] Abu-Rustum NR, Yashar CM, Bradley K, Campos SM, Chino J, Chon HS (2021). NCCN Guidelines® Insights: Uterine Neoplasms, Version 3.2021. J Natl Compr Canc Netw.

[4] Susumu N, Sagae S, Udagawa Y, Niwa K, Kuramoto H, Satoh S (2008). Randomized phase III trial of pelvic radiotherapy versus cisplatin-based combined chemotherapy in patients with intermediate- and high-risk endometrial cancer: a Japanese Gynecologic Oncology Group study. Gynecol Oncol.

[5] Kong A, Johnson N, Kitchener HC, Lawrie TA (2012). Adjuvant radiotherapy for stage I endometrial cancer. Cochrane Database Syst Rev.

[6] Lancellotta V, De Felice F, Vicenzi L, Antonacci A, Cerboneschi V, Costantini S (2020). The role of vaginal brachytherapy in stage I endometrial serous cancer: a systematic review. J Contemp Brachytherapy.

[7] Gómez-Raposo C, Merino Salvador M, Aguayo Zamora C, Casado SE (2020). Adjuvant chemotherapy in endometrial cancer. Cancer Chemother Pharmacol.

[8] de Boer SM, Powell ME, Mileshkin L, Katsaros D, Bessette P, Haie-Meder C (2018). Adjuvant chemoradiotherapy versus radiotherapy alone for women with high-risk endometrial cancer (PORTEC-3): final results of an international, open-label, multicentre, randomised, phase 3 trial. Lancet Oncol.

[9] Randall ME, Filiaci V, McMeekin DS, von Gruenigen V, Huang H, Yashar CM (2019). Phase III Trial: Adjuvant Pelvic Radiation Therapy Versus Vaginal Brachytherapy Plus Paclitaxel/Carboplatin in High-Intermediate and High-Risk Early Stage Endometrial Cancer. J Clin Oncol.

[10] Matei D, Filiaci V, Randall ME, Mutch D, Steinhoff MM, DiSilvestro PA (2019). Adjuvant Chemotherapy plus Radiation for Locally Advanced Endometrial Cancer. N Engl J Med.

[11] Squires BS, Quinn TJ, Nandalur SR, Jawad MS (2021). Adjuvant radiotherapy improves overall survival when added to surgery and chemotherapy for uterine carcinosarcoma: a surveillance, epidemiology, and end results analysis. Int J Clin Oncol.

[12] Kane LT, Fang T, Galetta MS, Goyal DKC, Nicholson KJ, Kepler CK (2020). Propensity score matching: a statistical method. Clin Spine Surg.

[13] Cole SR, Hernán MA (2008). Constructing inverse probability weights for marginal structural models. Am J Epidemiol.

[14] Austin PC, Stuart EA (2015). Moving towards best practice when using inverse probability of treatment weighting (IPTW) using the propensity score to estimate causal treatment effects in observational studies. Stat Med.

[15] Foreman J, Salim AT, Praveen A, Fonseka D, Ting DSW, Guang He M (2021). Association between digital smart device use and myopia: a systematic review and meta-analysis. Lancet Digit Health.

[16] Raffone A, Travaglino A, Raimondo D, Maletta M, De Vivo V, Visiello U (2022). Uterine carcinosarcoma vs endometrial serous and clear cell carcinoma: a systematic review and meta-analysis of survival. Int J Gynaecol Obstet.

[17] Altman AD, Ferguson SE, Atenafu EG, Köbel M, McAlpine JN, Panzarella T (2015). Canadian high risk endometrial cancer (CHREC) consortium: analyzing the clinical behavior of high risk endometrial cancers. Gynecol Oncol.

[18] Cham S, Huang Y, Tergas AI, Hou JY, Burke WM, Deutsch I (2017). Utility of radiation therapy for early-stage uterine papillary serous carcinoma. Gynecol Oncol.

[19] Nout RA, Smit VT, Putter H, Jürgenliemk-Schulz IM, Jobsen JJ, Lutgens LC (2010). Vaginal brachytherapy versus pelvic external beam radiotherapy for patients with endometrial cancer of high-intermediate risk (PORTEC-2): an open-label, non-inferiority, randomised trial. Lancet.

[20] Colombo N, Creutzberg C, Amant F, Bosse T, González-Martín A, Ledermann J (2016). ESMO-ESGO-ESTRO Consensus Conference on Endometrial Cancer: Diagnosis, Treatment and Follow-up. Int J Gynecol Cancer.

[21] Lin JF, Muñiz K, Sukumvanich P, Gehrig P, Beriwal S, Kelley JL (2016). Survival advantage associated with multimodal therapy in women with node-positive (stage-IIIC) uterine papillary serous carcinoma: a National Cancer Database study. BJOG.

[22] van Weelden WJ, Reijnen C, Eggink FA, Boll D, Ottevanger PB, van den Berg HA (2020). Impact of different adjuvant treatment approaches on survival in stage III endometrial cancer: a population-based study. Eur J Cancer.

[23] Xiang M, English DP, Kidd EA (2019). Defining the survival benefit of adjuvant pelvic radiotherapy and chemotherapy versus chemotherapy alone in stages III-IVA endometrial carcinoma. Gynecol Oncol.

[24] Lee LJ, Demaria R, Berkowitz R, Matulonis U, Viswanathan AN (2014). Clinical predictors of long-term survival for stage IVB uterine papillary serous carcinoma confined to the abdomen. Gynecol Oncol.

[25] Cuccu I, D'Oria O, Sgamba L, De Angelis E, GoliaD'Augè T, Turetta C (2023). Role of Genomic and Molecular Biology in the Modulation of the Treatment of Endometrial Cancer: Narrative Review and Perspectives. Healthcare (Basel).

[26] GoliaD'Augè T, Cuccu I, Santangelo G, Muzii L, Giannini A, Bogani G (2023). Novel Insights into Molecular Mechanisms of Endometrial Diseases. Biomolecules.

[27] Bogani G, Chiappa V, Lopez S, Salvatore C, Interlenghi M, D'Oria O (2022). Radiomics and Molecular Classification in Endometrial Cancer (The ROME Study): A Step Forward to a Simplified Precision Medicine. Healthcare (Basel).

