# Supplementary material for: The survival impact of adjuvant radiotherapy and chemotherapy in patients with non-endometrioid endometrial carcinomas: a PSM-IPTW analysis based on SEER database
Source: BMC Womens Health. 2023 May 20;23:278. doi: 10.1186/s12905-023-02429-6 (PMC10199615; doi:10.1186/s12905-023-02429-6)
Supplement: Supplementary file 2 — Additional file 2: Supplementary Table 1. Baseline characteristics before and after IPTW-adjusted by postoperative adjuvant treatment. Supplementary Table 2. Estimated five-year OS and CSS in endometrial clear cell carcinoma stratified by TNM stages and adjuvant treatments. [file 12905_2023_2429_MOESM2_ESM.docx]

Supplementary Table 1. Baseline characteristics before and after IPTW-adjusted by postoperative adjuvant treatment

|  | **CRT *vs.* RT alone** | | | | | | |  |  |  | **CRT *vs.* CT alone** | | | | | | | | | | | | | | |
| --- | --- | --- | --- | --- | --- | --- | --- | --- | --- | --- | --- | --- | --- | --- | --- | --- | --- | --- | --- | --- | --- | --- | --- | --- | --- |
|  | **Unadjusted** | | | **IPTW-adjusted** | | | |  |  |  | **Unadjusted** | | | | | | | |  |  | |  | **IPTW-adjusted** | | |
| Characteristics | CRT | RT alone | P value | CRT | RT alnoe |  | P value |  |  |  | CRT | | | | | | CT alone | P value | |  |  |  | CRT | CT | P value |
| Age group(years) |  |  |  |  |  |  |  |  |  |  |  | | | | | |  |  | |  |  |  |  |  |  |
| 50-65 | 1448(45.66) | 236(29.69) | 0.000** | 42.56% | 42.81% |  | 0.838 |  |  |  | 1448(45.66) | | | | | | 1543(43.50) | 0.080 | |  |  |  | 45.00% | 44.46% | 0.823 |
| <50 | 76(2.40) | 23(2.89) |  | 2.43% | 2.83% |  |  |  |  |  | 76(2.40) | | | | | | 108(3.04) |  | |  |  |  | 2.96% | 2.80% |  |
| >65 | 1647(51.94) | 536(67.42) |  | 55.01% | 54.36% |  |  |  |  |  | 1647(51.94) | | | | | | 1896(53.45) |  | |  |  |  | 52.04% | 52.74% |  |
| Year of diagnosis |  |  |  |  |  |  |  |  |  |  |  | | | | | |  |  | |  |  |  |  |  |  |
| 2004-2008 | 364(11.48) | 250(31.45) | 0.000** | 15.46% | 14.57% |  | 0.821 |  |  |  | 364(11.48) | | | | | | 537(15.14) | 0.000** | |  |  |  | 14.11% | 13.45% | 0.711 |
| 2009-2013 | 806(25.42) | 183(23.02) |  | 24.92% | 25.39% |  |  |  |  |  | 806(25.42) | | | | | | 1050(29.60) |  | |  |  |  | 27.94% | 27.87% |  |
| 2014-2018 | 2001(63.10) | 362(45.53) |  | 59.62% | 60.05% |  |  |  |  |  | 2001(63.10) | | | | | | 1960(55.26) |  | |  |  |  | 57.95% | 58.68% |  |
| Race recode |  |  |  |  |  |  |  |  |  |  |  | | | | | |  |  | |  |  |  |  |  |  |
| Black | 680(21.44) | 158(19.87) | 0.166 | 20.97% | 21.23% |  | 0.061 |  |  |  | 680(21.44) | | | | | | 778(21.93) | 0.002** | |  |  |  | 22.04% | 21.59% | 0.664 |
| Others* | 274(8.64) | 56(7.04) |  | 8.27% | 5.73% |  |  |  |  |  | 274(8.64) | | | | | | 395(11.14) |  | |  |  |  | 10.65% | 10.14% |  |
| White | 2217(69.91) | 581(73.08) |  | 70.75% | 73.05% |  |  |  |  |  | 2217(69.91) | | | | | | 2374(66.93) |  | |  |  |  | 67.31% | 68.26% |  |
| Marital status |  |  |  |  |  |  |  |  |  |  |  | | | | | |  |  | |  |  |  |  |  |  |
| Divorced/separated | 389(12.27) | 100(12.58) | 0.000** | 12.36% | 14.99% |  | 0.138 |  |  |  | 389(12.27) | | | | | | 467(13.17) | 0.002** | |  |  |  | 13.00% | 12.83% | 0.998 |
| Married | 1667(52.57) | 359(45.16) |  | 51.09% | 50.54% |  |  |  |  |  | 1667(52.57) | | | | | | 1743(49.14) |  | |  |  |  | 50.68% | 50.60% |  |
| Single/unmarried | 488(15.39) | 108(13.58) |  | 15.06% | 13.03% |  |  |  |  |  | 488(15.39) | | | | | | 613(17.28) |  | |  |  |  | 16.30% | 16.50% |  |
| Unknown | 166(5.23) | 30(3.77) |  | 4.82% | 3.82% |  |  |  |  |  | 166(5.23) | | | | | | 146(4.12) |  | |  |  |  | 4.66% | 4.61% |  |
| Widowed | 461(14.54) | 198(24.91) |  | 16.67% | 17.62% |  |  |  |  |  | 461(14.54) | | | | | | 578(16.30) |  | |  |  |  | 15.36% | 15.47% |  |
| Median household income |  |  |  |  |  |  |  |  |  |  |  |  |  |  |  |  |  |  |  |  |  |  |  |  |  |
| $50,000-65,000 | 886(27.94) | 216(27.17) | 0.246 | 27.35% | 26.60% |  | 0.250 |  |  |  | 886(27.94) | | | | | | 1033(29.12) | 0.265 | |  |  |  | 28.81% | 28.74% | 0.968 |
| <$50,000 | 359(11.32) | 107(13.46) |  | 11.93% | 10.00% |  |  |  |  |  | 359(11.32) | | | | | | 428(12.07) |  | |  |  |  | 11.88% | 11.70% |  |
| >$65,000 | 1926(60.74) | 472(59.37) |  | 60.72% | 63.39% |  |  |  |  |  | 1926(60.74) | | | | | | 2086(58.81) |  | |  |  |  | 59.32% | 59.56% |  |
| Grade |  |  |  |  |  |  |  |  |  |  |  |  |  |  |  |  |  |  |  |  |  |  |  |  |  |
| I | 49(1.55) | 13(1.64) | 0.011* | 1.57% | 2.47% |  | 0.316 |  |  |  | 49(1.55) | | | | | | 41(1.16) | 0.350 | |  |  |  | 1.23% | 1.32% | 0.969 |
| II | 116(3.66) | 50(6.29) |  | 4.06% | 4.68% |  |  |  |  |  | 116(3.66) | | | | | | 112(3.16) |  | |  |  |  | 3.45% | 3.41% |  |
| III | 1496(47.18) | 371(46.67) |  | 46.91% | 47.51% |  |  |  |  |  | 1496(47.18) | | | | | | 1656(46.69) |  | |  |  |  | 47.24% | 47.14% |  |
| IV | 769(24.25) | 169(21.26) |  | 23.61% | 21.70% |  |  |  |  |  | 769(24.25) | | | | | | 908(25.60) |  | |  |  |  | 25.36% | 24.85% |  |
| Unknown | 741(23.37) | 192(24.15) |  | 23.84% | 23.64% |  |  |  |  |  | 741(23.37) | | | | | | 830(23.40) |  | |  |  |  | 22.72% | 23.27% |  |
| Histology |  |  |  |  |  |  |  |  |  |  |  | | | | | |  |  | |  |  |  |  |  |  |
| Carcinosarcoma | 457(14.41) | 78(9.81) | 0.000** | 13.51% | 15.12% |  | 0.484 |  |  |  | 457(14.41) | | | | | | 424(11.95) | 0.000** | |  |  |  | 12.81% | 13.10% | 0.897 |
| Clear cell | 384(12.11) | 232(29.18) |  | 15.69% | 15.04% |  |  |  |  |  | 384(12.11) | | | | | | 361(10.18) |  | |  |  |  | 10.73% | 10.94% |  |
| Serous | 2330(73.48) | 485(61.01) |  | 70.80% | 69.85% |  |  |  |  |  | 2330(73.48) | | | | | | 2762(77.87) |  | |  |  |  | 76.46% | 75.96% |  |
| TNM stage |  |  |  |  |  |  |  |  |  |  |  | | | | | |  |  | |  |  |  |  |  |  |
| T1N0M0 | 1267(39.96) | 492(61.89) | 0.000** | 44.45% | 45.39% |  | 0.416 |  |  |  | 1267(39.96) | | | | | | 933(26.30) | 0.000** | |  |  |  | 32.50% | 32.67% | 0.998 |
| T2N0M0 | 306(9.65) | 139(17.48) |  | 11.26% | 10.83% |  |  |  |  |  | 306(9.65) | | | | | | 150(4.23) |  | |  |  |  | 6.73% | 6.73% |  |
| T3-4aN0MO | 415(13.09) | 72(9.06) |  | 12.17% | 10.18% |  |  |  |  |  | 415(13.09) | | | | | | 432(12.18) |  | |  |  |  | 12.45% | 12.70% |  |
| TanyN1M0 | 767(24.19) | 66(8.30) |  | 20.97% | 21.55% |  |  |  |  |  | 767(24.19) | | | | | | 539(15.20) |  | |  |  |  | 19.29% | 19.33% |  |
| TanyN2M0 | 150(4.73) | 4(0.50) |  | 3.88% | 3.24% |  |  |  |  |  | 150(4.73) | | | | | | 104(2.93) |  | |  |  |  | 3.81% | 3.86% |  |
| TanyNanyM1 | 266(8.39) | 22(2.77) |  | 7.28% | 8.82% |  |  |  |  |  | 266(8.39) | | | | | | 1389(39.16) |  | |  |  |  | 25.22% | 24.71% |  |
| Surgery mode |  |  |  |  |  |  |  |  |  |  |  | | | | | |  |  | |  |  |  |  |  |  |
| Extended hysterectomy | 276(8.70) | 67(8.43) | 0.804 | 8.70% | 8.68% |  | 0.988 |  |  |  | 276(8.70) | | | | | | 406(11.45) | 0.000** | |  |  |  | 9.74% | 10.05% | 0.670 |
| Total hysterectomy | 2895(91.30) | 728(91.57) |  | 91.30% | 91.32% |  |  |  |  |  | 2895(91.30) | | | | | | 3141(88.55) |  | |  |  |  | 90.26% | 89.95% |  |
| Lymphadenectomy |  |  |  |  |  |  |  |  |  |  |  | | | | | |  |  | |  |  |  |  |  |  |
| No | 362(11.42) | 140(17.61) | 0.000** | 12.51% | 13.20% |  | 0.800 |  |  |  | 362(11.42) | | | | | | 1156(32.59) | 0.000** | |  |  |  | 22.88% | 22.62% | 0.969 |
| SLN biopsy/removed | 225(7.10) | 38(4.78) |  | 6.62% | 6.11% |  |  |  |  |  | 225(7.10) | | | | | | 143(4.03) |  | |  |  |  | 5.27% | 5.31% |  |
| Yes | 2584(81.49) | 617(77.61) |  | 80.87% | 80.69% |  |  |  |  |  | 2584(81.49) | | | | | | 2248(63.38) |  | |  |  |  | 71.85% | 72.07% |  |
| Tumor Size(mm) |  |  |  |  |  |  |  |  |  |  |  | | | | | |  |  | |  |  |  |  |  |  |
| <20 | 377(11.89) | 123(15.47) | 0.000** | 12.59% | 12.94% |  | 0.998 |  |  |  | 377(11.89) | | | | | | 405(11.42) | 0.000** | |  |  |  | 11.25% | 11.60% | 0.941 |
| 20-39 | 721(22.74) | 178(22.39) |  | 22.73% | 23.14% |  |  |  |  |  | 721(22.74) | | | | | | 740(20.86) |  | |  |  |  | 22.07% | 21.80% |  |
| 40-59 | 677(21.35) | 163(20.50) |  | 21.23% | 21.43% |  |  |  |  |  | 677(21.35) | | | | | | 654(18.44) |  | |  |  |  | 20.69% | 20.04% |  |
| 60-79 | 350(11.04) | 72(9.06) |  | 10.67% | 10.64% |  |  |  |  |  | 350(11.04) | | | | | | 386(10.88) |  | |  |  |  | 11.28% | 11.04% |  |
| ≥80 | 336(10.60) | 53(6.67) |  | 9.81% | 9.48% |  |  |  |  |  | 336(10.60) | | | | | | 389(10.97) |  | |  |  |  | 10.84% | 10.75% |  |
| Unknown | 710(22.39) | 206(25.91) |  | 22.97% | 22.37% |  |  |  |  |  | 710(22.39) | | | | | | 973(27.43) |  | |  |  |  | 23.86% | 24.77% |  |

CT: chemotherapy. RT: radiotherapy. CRT: chemoradiotherapy. SLN: sentinel lymph node. Race Others*:American Indian, Asian/Pacific Islander. IPTW: inverse probability treatment weighting

Supplementary Table 2. Estimated five-year OS and CSS in endometrial clear cell carcinoma stratified by TNM stages and adjuvant treatments

|  |  | **Cause-specific survival** | | |  | **Overall survivial** | | |
| --- | --- | --- | --- | --- | --- | --- | --- | --- |
| TNM stage | Number | Estimate, % (95%CI) | Hazard ratio (95%CI) | P value |  | Estimate, % (95%CI) | Hazard ratio (95%CI) | P value |
| T1N0M0 | 373 | 85.71%(81.11-89.27) |  |  |  | 81.60%(76.67-85.59) |  |  |
| VBT alone | 71 | 80.29% (69.21-87.73) | Reference |  |  | 82.24% (73.53-88.31) | Reference |  |
| CT+EBRT | 39 | 83.87% (65.50-92.95) | 0.825 (0.287-2.376) | 0.722 |  | 83.87% (65.50-92.95) | 0.697 (0.249-1.957) | 0.494 |
| EBRT+VBT | 16 | 92.00% (55.31-98.83) | 0.422 (0.054-3.270) | 0.409 |  | 77.78% (45.77-92.24) | 1.070 (0.305-3.756) | 0.916 |
| CT alone | 81 | 87.10% (75.85-93.33) | 0.651 (0.262-1.618) | 0.355 |  | 84.13% (72.51-91.13) | 0.686 (0.301-1.565) | 0.371 |
| EBRT alone | 55 | 83.91% (69.19-91.98) | 0.833 (0.323-2.150) | 0.706 |  | 73.91% (58.66-84.26) | 1.206 (0.550-2.644) | 0.640 |
| CT+VBT | 95 | 88.41% (78.15-94.03) | 0.569 (0.229-1.416) | 0.226 |  | 85.71% (75.08-92.05) | 0.600 (0.263-1.368) | 0.224 |
| CT+EBRT+VBT | 16 | 91.67% (53.90-98.78) | 0.429 (0.055-3.319) | 0.417 |  | 91.67% (53.90-98.78) | 0.362 (0.047-2.770) | 0.328 |
| T2N0M0 | 109 | 75.14%(65.00-82.72) |  |  |  | 70.53%(60.25-78.61) |  |  |
| VBT alone | 12 | 78.95% (38.74-94.28) | Reference |  |  | 70.00% (32.87-89.19) | Reference |  |
| CT+EBRT | 14 | 90.91% (50.81-98.67) | 0.365 (0.033-4.032) | 0.411 |  | 90.91% (50.81-98.67) | 0.243 (0.025-2.341) | 0.221 |
| EBRT+VBT | 16 | 80.00% (49.98-93.07) | 0.896 (0.150-5.366) | 0.904 |  | 74.19% (45.01-89.44) | 0.789 (0.176-3.530) | 0.757 |
| CT alone | 14 | 66.67% (33.74-85.97) | 1.603 (0.293-8.757) | 0.586 |  | 60.00% (28.97-81.01) | 1.347 (0.322-5.639) | 0.684 |
| EBRT alone | 23 | 57.14% (33.80-74.92) | 2.406 (0.520-11.140) | 0.261 |  | 53.49% (30.89-71.66) | 1.770 (0.487-6.432) | 0.386 |
| CT+VBT | 15 | 76.92% (44.21-91.91) | 0.980 (0.164-5.870) | 0.982 |  | 76.92% (44.21-91.91) | 0.642 (0.129-3.181) | 0.587 |
| CT+EBRT+VBT | 15 | 90.91% (50.81-98.67) | 0.389 (0.035-4.295) | 0.441 |  | 82.61% (46.51-95.34) | 0.522 (0.087-3.124) | 0.476 |
| T3-4N0M0 | 114 | 60.00%(49.72-68.84) |  |  |  | 56.10%(45.96-65.06) |  |  |
| VBT alone | 3 | 33.33% (0.90-77.41) | Reference |  |  | 33.33% (9.90-77.41) | Reference |  |
| CT+EBRT | 22 | 55.00% (31.34-73.49) | 0.455 (0.098-2.116) | 0.315 |  | 55.00% (31.34-73.49) | 0.465 (0.100-2.159) | 0.328 |
| EBRT+VBT | 5 | 71.43% (8.97-95.41) | 0.294 (0.027-3.247) | 0.318 |  | 33.33% (2.71-71.57) | 0.912 (0.152-5.472) | 0.920 |
| CT alone | 48 | 53.66% (37.41-67.42) | 0.477 (0.111-2.053) | 0.320 |  | 51.81% (35.79-65.64) | 0.511 (0.119-2.192) | 0.366 |
| EBRT alone | 11 | 61.90% (27.53-83.67) | 0.373 (0.068-2.043) | 0.256 |  | 61.90% (27.53-83.67) | 0.381 (0.070-2.089) | 0.266 |
| CT+VBT | 16 | 79.31% (48.65-92.81) | 0.172 (0.029-1.036) | 0.055 |  | 73.33% (43.62-89.05) | 0.235 (0.043-1.289) | 0.095 |
| CT+EBRT+VBT | 9 | 73.33 % (28.74-92.58) | 0.235 (0.033-1.675) | 0.149 |  | 62.50% (22.93-86.07) | 0.359 (0.060-2.152) | 0.262 |
| TanyN1M0 | 210 | 51.86%(44.21-58.97) |  |  |  | 45.21%(37.87-52.24) |  |  |
| CT+EBRT | 67 | 58.93% (44.95-70.49) | Reference |  |  | 52.14% (38.69-63.97) | Reference |  |
| VBT alone | 1 | 0 |  |  |  | 0 |  |  |
| EBRT+VBT | 6 | 27.27% (2.44-63.48) | 1.677 (0.579-4.859) | 0.340 |  | 27.27% (2.44-63.48) | 1.395 (0.488-3.983) | 0.534 |
| CT alone | 72 | 52.07%(38.86-63.72) | 1.181 (0.683-2.041) | 0.552 |  | 44.88%(32.44-56.55) | 1.176 (0.716-1.934) | 0.522 |
| EBRT alone | 11 | 40.00% (12.27-67.02) | 1.999 (0.814-4.912) | 0.131 |  | 33.33% (9.13-60.39) | 1.948 (0.850-4.461) | 0.115 |
| CT+VBT | 20 | 50.00% (24.52-71.05) | 1.175 (0.525-2.631) | 0.694 |  | 41.18% (18.58-62.64) | 1.219 (0.591-2.512) | 0.592 |
| CT+EBRT+VBT | 33 | 45.10% (25.58-62.81) | 1.366 (0.701-2.661) | 0.360 |  | 39.62% (21.48-57.28) | 1.305 (0.704-2.418) | 0.397 |
| TanyNanyM1 | 171 | 22.22%(16.01-29.08) |  |  |  | 18.35%(12.77-24.75) |  |  |
| CT alone | 146 | 20.30% (13.95-27.50) | Reference |  |  | 16.79% (11.09-23.50) | Reference |  |
| CT+EBRT | 12 | 23.81% (4.74-50.91) | 0.869 (0.423-1.784) | 0.702 |  | 23.81% (4.74-50.91) | 0.811 (0.396-1.663) | 0.568 |
| CT+EBRT+VBT | 8 | 80.00%(20.38-96.92) | 0.125 (0.017-0.897) | 0.039 |  | 63.64%(16.06-89.43) | 0.232 (0.057-0.940) | 0.041 |
| EBRT alone | 1 | 0 | 18.487 (2.347-145.612) | 0.006 |  | 0 | 16.588 (2.127-129.389) | 0.007 |
| CT+VBT | 3 | 0 | 1.856 (0.587-5.865) | 0.292 |  | 0 | 1.752 (0.555-5.530) | 0.339 |
| VBT alone | 1 | 0 | 0.000 (0.000-Inf) | 0.995 |  | 0 | 0.541 (0.075-3.887) | 0.541 |

CT:chemotherapy. EBRT: external beam radiotherapy. VBT: vaginal brachytherapy
